# Potentially Functional SNPs (pfSNPs) as Novel Genomic Predictors of 5-FU Response in Metastatic Colorectal Cancer Patients

**DOI:** 10.1371/journal.pone.0111694

**Published:** 2014-11-05

**Authors:** Jingbo Wang, Xu Wang, Mingjue Zhao, Su Pin Choo, Sin Jen Ong, Simon Y. K. Ong, Samuel S. Chong, Yik Ying Teo, Caroline G. L. Lee

**Affiliations:** 1 Department of Biochemistry, Yong Loo Lin School of Medicine, National University of Singapore, Singapore, Singapore; 2 Saw Swee Hock School of Public Health, National University of Singapore, Singapore, Singapore; 3 Division of Medical Oncology, National Cancer Centre, Singapore, Singapore; 4 Department of Paediatrics, Yong Loo Lin School of Medicine, National University of Singapore, Singapore, Singapore; 5 Division of Medical Sciences, National Cancer Centre, Singapore, Singapore; 6 Duke-NUS Graduate Medical School, Singapore, Singapore; Queen Mary Hospital, Hong Kong

## Abstract

5-Fluorouracil (5-FU) and its pro-drug Capecitabine have been widely used in treating colorectal cancer. However, not all patients will respond to the drug, hence there is a need to develop reliable early predictive biomarkers for 5-FU response. Here, we report a novel potentially functional Single Nucleotide Polymorphism (pfSNP) approach to identify SNPs that may serve as predictive biomarkers of response to 5-FU in Chinese metastatic colorectal cancer (CRC) patients. 1547 pfSNPs and one variable number tandem repeat (VNTR) in 139 genes in 5-FU drug (both PK and PD pathway) and colorectal cancer disease pathways were examined in 2 groups of CRC patients. Shrinkage of liver metastasis measured by RECIST criteria was used as the clinical end point. Four non-responder-specific pfSNPs were found to account for 37.5% of all non-responders (P<0.0003). Five additional pfSNPs were identified from a multivariate model (AUC under ROC = 0.875) that was applied for all other pfSNPs, excluding the non-responder-specific pfSNPs. These pfSNPs, which can differentiate the other non-responders from responders, mainly reside in tumor suppressor genes or genes implicated in colorectal cancer risk. Hence, a total of 9 novel SNPs with potential functional significance may be able to distinguish non-responders from responders to 5-FU. These pfSNPs may be useful biomarkers for predicting response to 5-FU.

## Introduction

Every year, more than one million individuals worldwide will develop colorectal cancer [1], accounting for 10% of the global cancer burden. Colorectal cancer (CRC) is the most frequently diagnosed cancer in Singapore (7,909 new cases between 2005–2009) [2]. More than half of CRC patients develop metastatic disease (stage 4) either at diagnosis or at relapse following initial curative intent therapy. This translates to a substantial proportion of patients who may need treatment for the metastasis or relapse of colorectal cancer.

5-fluorouracil (5-FU) and its pro-drug, Capecitabine, are widely used in treating CRC. It has been proposed that there are two distinct modes of action for 5-FU. First, it acts as anti-metabolite whereby its active form, FdUMP, produced by Thymidine Phosphorylase (TYMP), inhibits Thymidylate Synthase (TYMS). Second, it can induce cell death, whereby incorporation of its active products FUTP and FdUTP into RNA and DNA, respectively, leads to subsequent cell apoptosis [3]. Uridine Monophosphate Synthetase (UMPS, also known as OPRT) is responsible for converting 5-FU to FUMP, which is the first step of producing FUTP and FdUTP. However, the two pathways may overlap, because the intermediate product in the “cell toxicity” pathway, FUDP, may also be converted to FdUDP and subsequently FdUMP and participate in the “anti-metabolite” pathway. 5-FU is catabolized into the inactive form of DHFU by Dihydropyrimidine Dehydrogenase (DPYD), and DPYD is the rate-limiting enzyme in degrading 5-FU.

At present, there are no reliable tests for early prediction of response to 5-FU. Developing a reliable early predictive biomarker of response to common chemotherapy, like 5-FU, in metastatic colorectal cancer has the potential to lead to appropriate tailoring of treatment for individual patients and help move us closer to a truly personalized care. Overall economic cost benefits are realized in both predicted responders and non-responders. Responders get appropriate treatment with confidence of anticipated response. Predicted non-responders to conventional treatment avoid wasted expense of 3 cycles of futile treatment, unnecessary toxicities that themselves require remedies and time loss in terms of futile treatment and loss of a window of opportunity for effective treatment. These patients may be selected as candidates for novel therapies and combination chemotherapy.

Thus far, only variants in the “5-FU PD” genes were reported to be significantly associated with 5-FU efficacy measured by tumour shrinkage in some studies [4]–[7]. Unfortunately, replication of such reported association between “5-FU PD” gene variants and tumour shrinkage remains challenging [4], [6], [8]–[13], suggesting the possible presence of other loci in determining 5-FU efficacy.

It was interesting to note that the variants in the “5-FU PK” genes were mainly investigated for their association with 5-FU toxicity, not efficacy [14]–[17], despite the expression levels of these genes having been previously associated with 5-FU efficacy [18]. Limited efforts [8], [19] attempted to explore the possible association for efficacy, but failed.

Furthermore, most studies on response to 5-FU treatment focused primarily on SNPs in a few candidate genes. Only one study examined 21 variants primarily in coding region of 11 genes involved in metabolism/action of 5-FU and other related pharmacological pathways [19]. However, this study still does not comprehensively interrogate all possible variants that may be involved in 5-FU response.

Another limitation of current studies is that only univariate analyses have, thus far, been employed and this may not have sufficient power for detection of association of drug response with less common/rare SNPs with small sample size. Multivariate model was successfully employed to estimate the appropriate dose of warfarin based on clinical and genetic data [20].

Hence, in this study, we employ a novel approach interrogating potentially functional SNPs (pfSNPs) in relevant drug and disease pathway to identify association with drug response. 1,547 potentially functional SNPs+1 VNTR (Variable Number Tandem Repeat) from 139 genes in the drug (both PK and PD pathway) and disease pathways were examined. Potentially functional SNPs were identified using the pfSNP Web Resource (http://pfs.nus.edu.sg/) [21] which included SNPs that were previously reported to be functional or associated with disease/drug response; SNPs that were inferred to be potentially functional from genetic approaches as well as those predicted to be potentially functional from sequence motifs.

As the number of samples was limited, a two-step study design was employed. In the first stage, we examined 62 patients who were only on Capecitabine, a pro-drug of 5-FU, to identify interesting SNPs that are marginally associated with drug response as measured by tumor shrinkage. These SNPs were then examined in another group of 27 patients who were treated with 5-FU and oxaliplatin. Combined Multivariate and Collapsing (CMC) analysis was employed to evaluate the less common (≤5%), non-responder-specific pfSNPs for their association with 5-FU drug response while a logistic regression based multivariate model using stepwise Akaike Information Criterion (stepAIC) procedure was used to interrogate the other pfSNPs for their association with 5-FU drug response in patients that do not carry the non-responder-specific pfSNPs.

## Materials and Methods

### Patient samples and clinical parameters

The shrinkage of liver metastatic CRC tumour was used as the clinical endpoint for measuring treatment efficacy. Response Evaluation Criteria In Solid Tumours (RECIST) [22] is used to determine tumour response. In the patients recruited, there was no patient belonging to the ‘Complete Response’ category. Patients with ‘Partial Response’ were deemed as ‘Responders’ and patients with ‘Progressive Disease’ or ‘Stable Disease’ were classified as ‘Non-Responders’ in the association analysis.

A total of 89 unrelated Chinese metastatic CRC patients were recruited. All patients had liver metastasis and were given neo-adjuvant chemotherapy prior to operation for the liver lesion. **Table 1** shows the characteristics of the patients in each study.

**Table 1:** The demographic characteristics of the patients recruited for each study.

	Group 1	Group 2
**Number of Patients**	62	27
**Ages (Median)**	36–78 (60)	42–86 (62)
**Males (Females)**	49(13)	18(9)
**Prior Drug Exposure**		
5-FU alone	0	2
Capecitabine alone	0	3
5-FU + oxaliplatin	0	2
Capecitabine + oxaliplatin	0	0
5-FU + Radio Therapy	0	2
**Drugs Treated**		
5-FU alone	0	2
Capecitabine alone	62	1
5-FU + oxaliplatin	0	15
Capecitabine + oxaliplatin	0	8
5-FU + Irinotecan	0	1
**Response**		
Partial response	13	12
Stable disease	31	7
Progressive disease	18	8

In group 1, 62 unrelated Stage IV CRC Chinese patients were recruited. These patients were treated with only Capecitabine and they have never been previously exposed to this drug. Of these 62 patients, 13 had partial response, 31 had stable disease, and 18 had progressive disease. Hence, the response rate of this group of patients is only ∼21%, which is typical for single-agent treatment [23]. ∼79% of the patients were male, and the median age of the cohort was 60 years.

In group 2, 27 unrelated Chinese liver metastatic CRC patients who were treated with 5-FU (a few had Capecitabine) alone (a few) or with oxaliplatin regime (most) as their neo-adjuvant chemotherapy were examined. Some of these patients had also been previously exposed to these drugs. Of these 27 patients, 12 had partial response, seven had stable disease, and eight had progressive disease. Hence, the response rate was ∼45%, which is typical for patients undergoing 2-drug combination therapy [24]. Two-thirds of these patients are males, and had a median age of 62 years.

### Ethics statement

This study has been approved by Singhealth Centralized Institutional Review Board (CIRB) (Reference No: NC05–22 and 2005/421/B).

### Selection of potentially functional SNPs (pfSNPs) for association study

The pfSNP resource (http://pfs.nus.edu.sg/) [21] was employed to identify SNPs in genes associated with 5-FU/Capecitabine, oxaliplatin as well as colorectal cancer. Approximately 2800 pfSNPs in 214 genes were found to be associated with keywords including “fluorouracil”, “5-fluorouracil”, “capecitabine”, “platinum”, “oxaliplatin” as well as “colorectal cancer”. As only 1,536 SNPs can be genotyped within a single customized GoldenGate Genotyping Array (Illumina, Inc), the following criteria were employed to select a subset of these 2800 pfSNPs: all SNPs within the promoter, coding, 5′/3′ un-translated regions which has a GoldenGate Score (GGS: measure of assay quality by their platform) of greater than 0.5 were selected. For the introns, pfSNP with a GGS>0.7 were selected and monomorphic ones reported in HapMap CHB population were excluded, except for those previously reported as functional. For any adjacent SNPs that may interfere with each other in the assay, we selected the SNP according to the following order: “Previously reported → Non-Synonymous → Synonymous → UTR → Intron”. A list of all of the SNPs included on the GoldenGate array is available as **Table S1**.

Among the markers that were unsuitable to be genotyped by GoldenGate assay, 14 important markers in 5-FU response prediction were selected to be genotyped by other methods (listed in **Table S2**). The 14 markers include the previously well studied VNTR with embedded SNP [25] as well as the 6bps 3′ UTR indel in the TYMS gene because GoldenGate technology could only genotype SNPs. Other markers are SNPs with low GoldenGate scores in the TYMS, TYMP and DPYD genes. A customized Sequenom’s MassARRAY panel was used to genotype 11 of the 14 markers and 1 SNP was genotyped by ABI TaqMan. In addition, we developed a novel method to genotype the VNTR (rs2853542) and embedded SNP (rs34743033) in the TYMS gene. This VNTR can have either 2 or 3 repeats, and the SNP can occur in both the second and third repeat [26]. We developed a robust method using Sanger Sequencing for this purpose. A fragment of 486 bp was amplified using the primers (F- CTGCTGGCTTAGAGAAGGCG and R- AGCGGAGGATGTGTTGGATC) and the amplicon was sequenced in both directions using the forward and reverse primers. Different genotypes would yield distinct patterns on the forward and reverse sequencing reads (As shown in **Figure S1**), allowing the genotype to be easily deduced.

In summary, there were 1,536 markers (Listed in **Table S1**) genotyped with a single customized Illumina GoldenGate SNP genotyping array, 11 (Listed in **Table S2**) by Sequenom’s MassARRAY, 1 (rs11479) by ABI TaqMan and the VNTR (rs2853542), with the embedded SNP (rs34743033) was genotyped using Sanger Sequencing.

The distribution of the SNPs and genes selected in the three categories (CRC, Fluorouracil and Platinum related) is depicted in **Figure S2**. More than half of the SNPs (863) are from fluorouracil-related genes and 702 SNPs are from platinum-related genes. A considerable portion of the SNPs (656) are from CRC-related genes, although 40% (32 out of 80) of these genes and 88% (579 out of 656) of the SNPs are related to fluorouracil and/or platinum as well.

The numbers of SNPs in each gene region and function category covered in this study are shown in **Figure S3**. Each of the four gene regions, namely promoter, coding, intron and 3′ UTR, are adequately covered in general. For promoter and 3′UTR, most of the SNPs genotyped are those that change TF binding sites. In the coding region, SNPs that change ESE/ESS sites are the most abundant, and non-synonymous SNPs that cause deleterious effects are the second most abundant. The intron region SNPs are enriched with those with a signature of recent positive selection.

The distribution of SNP minor allele frequency for the intronic versus non-intronic region is shown in **Table S3**. For coding, 3′UTR, and promoter regions, a number of SNPs not previously genotyped by HapMap has been genotyped in this study. As the study aims to also explore rare variants, a number of SNPs reported by HapMap to be monomorphic was also genotyped. For the intron region, since most of the SNPs are those with a signature of recent positive selection, they have MAF more than 5%. We did not genotype many monomorphic ones within introns.

### Single marker association analysis

Hardy-Weinberg equilibrium and minor allele frequency were analysed using the Microsoft Excel-based SNP Statistics Calculator developed in-house. Single marker association analysis was performed using PLINK [27]. The P-value was calculated by the permutation-based method, and the Odds Ratio (OR) and corresponding 95% confidence interval were determined using regular allele-based association analyses, because the permutation-based method does not provide such information. The genotype of the VNTR (rs2853542) and embedded SNP (rs34743033) in the TYMS gene is re-coded as a bi-allelic SNP comprising the high-expression allele and the low-expression allele according to Kawakami et al [26].

### Combined Multivariate and Collapsing (CMC) analyses for non-responder-specific SNPs

Combined Multivariate and Collapsing (CMC) method was first proposed as a method for analysing rare SNPs [28]. CMC utilizes Hotelling’s T^2^ test to analyse more than 2 groups of collapsed variants. When only 2 groups of such variants are analysed, Fisher’s exact test can be used. In this study, we used the Fisher’s exact test to evaluate if the collapsed minor allele of non-responder specific SNPs which are less common (≤5% minor allele frequency) would be a good indicator of responsiveness to 5-FU.

### Logistic regression based multivariate model using Akaike Information Criterion (stepAIC) procedure to predict drug response in patients who do not have non-responder-specific SNPs

Patients, who do not have any of the non-responder specific SNPs, were divided into 2 groups. Data from the first group comprising 80% of the patients was used to train a logistic regression based multivariate model by using the Akaike’s Information Criterion (AIC) in a stepwise algorithm (using R package “stepAIC”) while the data from the other 20% of patients were used to validate the model. The selected model was evaluated by the Area Under the Curve (AUC) of the Receiver Operating Characteristic (ROC) curves. The optimal cut-off point for the logistic regression was chosen where the maximum sum of sensitivity and specificity is obtained [29], [30].

## Results and Discussion

A high concordance (R^2^ = 0.9494) was observed between allele frequencies of SNPs in our study and those from the CHB (Chinese in Beijing) population in HapMap (Release 27) (**Figure S4**) affirming the quality of our genotyping. A large proportion of the SNPs examined in this study were either monomorphic (36%) or had a high minor allele frequency (MAF≥0.1, 46%) (**Figure S5**). Seventy-two and 66 SNPs in Groups 1 and 2, respectively, were found to significantly deviate from Hardy-Weinberg Equilibrium and were excluded from further analysis.

Genotype was successfully (97–100%) assigned in 9 out of 11 SNPs genotyped using the Sequenom’s MassARRAY. Sanger sequencing successfully assigned genotype of the 2 SNPs to all the samples while TaqMan assay successfully assigned genotypes to 96% of the samples. All the SNPs successfully genotyped by these methods were found to be in Hardy-Weinberg equilibrium.

### Single SNP association analysis identified three non-responder specific SNPs in the UMPS gene that may represent potential predictive biomarker for non-response to 5-FU

As the number of samples in this study was small, a cross validation approach was employed where the samples were segregated into 2 distinct groups for discovery and validation to enhance the robustness of our findings.

A total of 36 SNPs in 12 genes were found to be associated with drug response before multiple test correction in Group 1 patients (**Table 2**). As the sample size was small (n = 62), none of these markers were statistically significant after Bonferroni correction.

**Table 2:** List of SNPs showing P<0.05 in Group 1 ranked by P value.

							Allele Count				
SN	Gene	mRNA Location	AA Change	rs No.	Function Summary	P	NR (98)	Rsp (26)	OR	OR 95 CI	
1	ATP7A	E23/3UTR/T1960C	–	rs1062472	RPS	**0.003**	22	14	4.03	1.55	9.29
2	RRM1	5UR//A-2723G	–	rs3750996	TF	**0.004**	30	1	11.36	1.46	86.66
3	DLG5	E/23/G4442T	P1481Q	rs2289310	Reported,Non-Syn,ProteinDomain,Del AA, ESE/ESS	**0.005**	15	11	4.06	1.53	10.13
4	RRM1	5UR//T-265G	–	rs1735068	TF	**0.010**	19	12	3.56	1.26	7.70
5	RRM1	5UR//A-4023G	–	rs3794050	TF	**0.012**	20	12	3.34	1.26	7.70
6	RRM1	5UR//T-659C	–	rs1662162	TF	**0.012**	20	12	3.34	1.20	7.24
7	UMPS	5UR//T-1256A	–	rs12492095	TF	**0.013**	11	9	4.19	1.45	10.88
8	UMPS	E/3/G638C	–	rs1801019	Reported,Non-Syn,ESE/ESS	**0.013**	11	9	4.19	1.45	10.88
9	APC	I/2/C-230A	–	rs2464805	RPS, ISRE	**0.018**	6	6	4.60	1.40	16.42
10	SMARCA2	I/28/G-8275T|I/29/G2824T	–|–	rs7048976	RPS	**0.021**	39	4	3.64	1.09	10.62
11	RRM1	5UR//G-2528A	–	rs1561876	TF	**0.023**	21	12	3.14	1.20	7.24
12	RRM1	E/19/G2232A	A744A	rs1042858	ESE/ESS	**0.023**	21	12	3.14	1.14	6.83
13	RRM1	E19/3UTR/C316A	–	rs1042927	miRNA	**0.023**	21	12	3.14	1.14	6.83
14	WDR7	I/13/A323G	–	rs11664579	Reported	**0.024**	10	8	3.91	1.30	10.42
15	TFRC	E/4/C424T	S142G	rs3817672	Reported,Non-Syn,ProteinDomain	**0.025**	14	9	3.18	1.24	8.91
16	ATP7A	E/10/G2299C	V767L	rs2227291	Non-Syn,ProteinDomain,Del AA, ESE/ESS	**0.025**	22	12	2.96	1.14	6.83
17	ATP7A	I/12/C-882A	–	rs17139617	RPS	**0.025**	22	12	2.96	1.14	6.83
18	ABCC4	3DR//A75075G|E31/3UTR/A879G	–|–	rs1059751	Reported	**0.025**	38	17	2.98	1.19	7.20
19	ABCC4	3DR//A74234C|E31/3UTR/A38C	–|–	rs3742106	miRNA	**0.029**	40	17	2.74	1.10	6.63
20	ABCC4	3DR//T74507C|E31/3UTR/T311C	–|–	rs4148551	Reported	**0.029**	40	17	2.74	1.10	6.63
21	APC	E/16/T5465A	V1822D	rs459552	Reported,Non-Syn,ProteinDomain, ESE/ESS	**0.033**	5	5	4.43	1.23	17.40
22	APC	I/6/T-3774C	–	rs2431238	RPS	**0.033**	5	5	4.43	1.23	17.40
23	CDC2	5UR//C-3953T|I/1/C263T	–|–	rs2448341	TF, ISRE	**0.039**	42	5	3.15	1.07	8.76
24	RRM1	5UR//C-3890T	–	rs7934581	TF	**0.043**	13	8	2.91	0.95	6.98
25	ERCC6	I/6/T871G	–	rs4253101	RPS	**0.045**	50	7	2.83	1.14	7.59
26	SLCO6A1	I/10/A-2493G	–	rs1562961	RPS	**0.046**	38	16	2.53	1.02	6.01
27	SLCO6A1	I/11/C702T	–	rs10062613	RPS	**0.046**	38	16	2.53	1.02	6.01
28	SLCO6A1	I/12/A7020C	–	rs6873738	RPS	**0.046**	38	16	2.53	1.02	6.01
29	SLCO6A1	I/12/G-738T	–	rs6877722	RPS	**0.046**	38	16	2.53	1.02	6.01
30	SLCO6A1	I/12/T517C	–	rs1901512	RPS	**0.046**	38	16	2.53	1.02	6.01
31	SLCO6A1	I/3/T142C	–	rs10041525	RPS, ISRE	**0.046**	38	16	2.53	1.02	6.01
32	SLCO6A1	I/3/T220C	–	rs10041507	RPS, ISRE	**0.046**	38	16	2.53	1.02	6.01
33	SLCO6A1	I/4/A-9G	–	rs11746217	RPS, ISRE	**0.046**	38	16	2.53	1.02	6.01
34	SLCO6A1	I/6/A4781G	–	rs1452057	RPS	**0.046**	38	16	2.53	1.02	6.01
35	SLCO6A1	I/9/G1986T	–	rs1901521	RPS	**0.046**	38	16	2.53	1.02	6.01
36	SLCO6A1	I/9/T5068G	–	rs1901522	RPS	**0.046**	38	16	2.53	1.02	6.01

Abbreviations: Reported: previously reported in the literature to be associated with disease/function; ESE/ESS: change Exon splice enhancer/silencer; NMD: mRNA nonsense mediated decay; Non-syn: non-synonymous SNP; ProteinDomain: residing in important protein domains; miRNA: change miRNA binding site; RPS: show signature of recent positive selection; TF: change transcription factor binding site; ISRE: change intron splice regulatory element.

Nonetheless, there were 68 low frequency pfSNPs in Group 1 that were uniquely found only in non-responders (non-responder-specific pfSNP). To evaluate if any of these non-responder-specific pfSNPs may represent potential predictive biomarker for non-response to 5-FU, we examined a second group of 27 patients. Of these 68 non-responder specific pfSNPs, 24 remained non-responder-specific even in Group 2 and these are presented in **Table 3**. However, only 3 of the non-responder-specific SNPs in the Uridine Monophosphate Synthetase (UMPS) gene, namely rs2291078 (E/4/T1050A C350*), rs3772809 (E/6/A1336G H446Y) and rs3772810 (E6/3UTR/A28G) (**Table 2** **,** SNPs 42–44) (**Table 3**, SNPs 1–3) were found to be statistically significant (p = 0.036 before multiple test correction) in Group 2. When the 2 groups of patients were combined and analysed, these 3 SNPs remained statistically significant (p = 0.032 before multiple test correction). The observation that out of a total of 89 patients in the combined group, no responders were found to carry this allele suggests that this allele may be a “causal” allele for determining the response to 5-FU. The non-statistical significant data obtained (after multiple test correction) suggests that this may not be the only “causal” alleles for 5-FU response and there are likely other alleles that also play a role in 5-FU response suggesting “locus heterogeneity” of response to 5-FU.

**Table 3:** List of SNPs with non-responder specific allele in both groups.

					MAF			Group 1			Group 2			Combined		
									Allele Count			Allele Count			Allele Count	
SN	Gene	mRNA Location	AA Change	rs No.	HapMap	This study	Function Summary	P	NR (98)	Rsp (26)	P	NR (30)	Rsp (24)	P	NR (128)	Rsp (50)
1	UMPS	E/4/T1050A	C350*	rs2291078	–	7.3%	NMD, Non-Syn,ProteinDomain,ESE/ESS	0.140	8	0	**0.036**	5	0	**0.032**	13	0
2	UMPS	E/6/A1336G	H446Y	rs3772809	5.4%	7.3%	Non-Syn,ProteinDomain,ESE/ESS	0.140	8	0	**0.036**	5	0	**0.032**	13	0
3	UMPS	E6/3UTR/A28G	–	rs3772810	5.4%	7.3%	miRNA, TF	0.140	8	0	**0.036**	5	0	**0.032**	13	0
4	TK1	5UR//G-1181A	–	rs8071253	–	3.4%	TF	0.250	5	0	0.367	1	0	0.119	6	0
5	DPYD	I/3/A22444C	–	rs10493895	1.8%	2.8%	Reported	0.305	4	0	0.367	1	0	0.156	5	0
6	DPYD	I/3/A39932G	–	rs10747486	1.9%	2.8%	RPS	0.305	4	0	0.367	1	0	0.156	5	0
7	DPYD	I/3/C30060T	–	rs1931063	2.2%	2.8%	RPS	0.305	4	0	0.367	1	0	0.156	5	0
8	DPYD	I/3/G-27495C	–	rs4537601	1.8%	2.8%	RPS	0.305	4	0	0.367	1	0	0.156	5	0
9	DPYD	I/3/G29092A	–	rs1333717	1.8%	2.8%	RPS	0.305	4	0	0.367	1	0	0.156	5	0
10	WDR7	I/1/A35G	–	rs501415	2.3%	2.8%	TF, ISRE	0.305	4	0	0.367	1	0	0.156	5	0
11	DPYD	I/4/A5229G	–	rs6683957	0.7%	2.2%	RPS	0.376	3	0	0.367	1	0	0.206	4	0
12	DPYD	I/4/G3787C	–	rs4970728	2.2%	2.2%	RPS	0.376	3	0	0.367	1	0	0.206	4	0
13	REV3L	E/26/C8285T	R2762Q	rs3218592	2.9%	2.2%	Non-Syn,ProteinDomain,DelAA, ESE/ESS	0.376	3	0	0.367	1	0	0.206	4	0
14	WDR7	E27/3UTR/C112T|E28/3UTR/C112T	–|–	rs3745032	–	2.2%	TF	0.376	3	0	0.367	1	0	0.206	4	0
15	WDR7	E27/3UTR/C2176G|E28/3UTR/C2176G	–|–	rs3745030	3.3%	2.2%	TF	0.376	3	0	0.367	1	0	0.206	4	0
16	WDR7	I/14/T9751A	–	rs11876256	2.2%	2.2%	RPS	0.376	3	0	0.367	1	0	0.206	4	0
17	WDR7	I/19/T-30795A|I/20/T-30795A	–|–	rs2576415	2.2%	2.8%	RPS	0.376	3	0	0.197	2	0	0.156	5	0
18	WDR7	I/12/C-2229T	–	rs11877604	2.2%	1.7%	RPS	0.467	2	0	0.367	1	0	0.271	3	0
19	SMARCD1	E/4/A423G	V141V	rs2307083	0.0%	1.1%	CodonDiff, TF	0.612	1	0	0.367	1	0	0.374	2	0
20	UPB1	5UR//G-96A	–	rs2232861	–	1.1%	TF	0.612	1	0	0.367	1	0	0.374	2	0
21	WDR7	I/18/A-2783C|I/19/A-2783C	–|–	rs9946253	0.4%	1.1%	Reported	0.612	1	0	0.367	1	0	0.374	2	0
22	WDR7	I/20/A20363G|I/21/A20363G	–|–	rs2083020	0.4%	1.1%	Reported	0.612	1	0	0.367	1	0	0.374	2	0
23	WDR7	I/21/A532G|I/22/A532G	–|–	rs8094838	0.4%	1.1%	Reported	0.612	1	0	0.367	1	0	0.374	2	0
24	WDR7	I/21/T-2912G|I/22/T-2912G	–|–	rs6566846	0.0%	1.1%	Reported, RPS	0.612	1	0	0.367	1	0	0.374	2	0

Abbreviations: Reported: previously reported in the literature to be associated with disease/function; ESE/ESS: change Exon splice enhancer/silencer; NMD: mRNA nonsense mediated decay; Non-syn: non-synonymous SNP; ProteinDomain: residing in important protein domains; miRNA: change miRNA binding site; RPS: show signature of recent positive selection; TF: change transcription factor binding site; ISRE: change intron splice regulatory element; DelAA: Deleterious amino acid change; CodonDiff: High codon usage difference.

The UMPS gene is important in determining 5-FU response, as it converts 5-FU into its active metabolite, FUMP, which can participate in both the cell toxicity pathway as well as in the “anti-metabolite” pathway. In the cell toxicity pathway, FUMP can be further converted into FUTP and FdUTP which is then incorporated into RNA and DNA respectively. In the “anti-metabolite” pathway, FUMP can be converted into FdUMP and inhibits TYMS.

**Figure 1:**
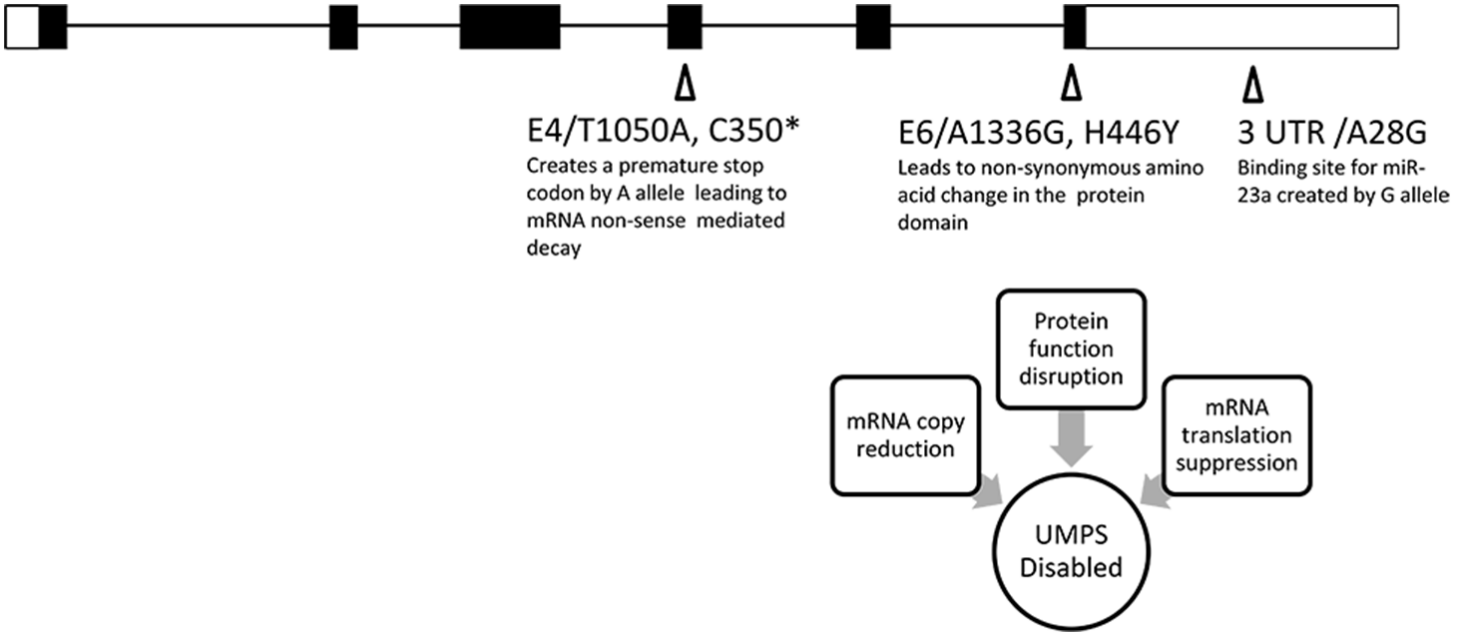
The molecular functions of the three SNPs in UMPS gene with minor allele uniquely found in non-responders are all linked to disabling UMPS.

These 3 alleles in the UMPS gene are in perfect linkage disequilibrium (LD) and hence will occur together all the time. The predicted molecular functions of the three alleles unique to the non-responders are all associated with the disruption of the UMPS gene function and support their unique presence in the non-responders (**Figure 1**).

The A allele of rs2291078 (UMPS E/4/T1050A C350*) was predicted to create a stop codon in exon 4 of the UMPS mRNA. UMPS mRNA containing this stop codon may be quickly degraded since stop codon appearing more than 50 bps from the last exon-exon junction would induce non-sense mediated decay of mRNA [31]. Therefore, patients carrying this A allele may have lower UMPS mRNA and protein abundance.

The co-occurrence of 3′UTR pfSNP rs3772810 (UMPS E6/3UTR/A28G) with this pfSNP rs2291078 suggests that the expression of this gene may be further attenuated. The G allele of the 3′UTR SNP rs3772810 (UMPS E6/3UTR/A28G) is predicted to create binding sites for miRNA 23a, 23b and 130a*. The miRNA 23a is shown to be up-regulated under hypoxic condition commonly found in tumors [32]. Notably, a recent publication reported that miRNA 23a is up-regulated in metastatic colorectal cancer [33] suggesting that patients with the G allele may be non-responsive to 5-FU since miRNA 23a may suppress the expression of UMPS mRNA containing the G allele.

Also co-occurring with these 2 pfSNPs, is the non-synonymous pfSNP rs3772809 (UMPS E/6/A1336G H446Y) which have the potential to alter the function of the UMPS gene. Hence, these 3 co-occurring pfSNPs, which accounted for 17.2% of all the non-responders have the potential to be the causal variants affecting the function of UMPS and thus response to 5-FU although further experiments are required to validate the potential functionality of these pfSNPs.

### Combined Multivariate and Collapsing (CMC) analyses revealed that a minimum of four non-responder specific pfSNPs that are not in linkage equilibrium can significantly distinguish non-responders from responders

We proceeded to determine if combination of non-responder specific pfSNPs (**Table 3**) can account for a greater percentage of 5-FU non-responders than the abovementioned 3 non-responder specific UMPS pfSNPs in perfect LD that show statistical significance (before multiple test corrections). Since the 3 non-responder specific UMPS pfSNPs are in perfect LD, only one was selected for further analyses. We then identify the minimum number of additional non-responder specific pfSNPs from **Table 3** that can account for the maximum percentage of 5-FU non-responders and employed Combined Multivariate and Collapsing (CMC) analyses [28] to determine its statistical significance.

Notably, three other non-responder specific pfSNPs together with any one of the UMPS non-responder specific pfSNPs were found to account for 37.5% of all non-responders from the 2 groups of patients (**Table 4**). CMC analyses revealed statistical significance (P = 0.0003) of these 4 non-responder specific pfSNPs (3 non UMPS plus any one of the 3 UMPS pfSNPs) suggesting significant association of these pfSNPs with non-responsiveness.

**Table 4:** The list of less-common SNPs with non-responder specific allele and their predicted molecular functions.

S.N	rsNo	Gene Name	mRNA Location	AA Change	Non-responder Specific Allele and Frequency (HapMap/This Study)	Allele Count		P	Potential Molecular Function
						NR	Rsp		
1	rs3772810	UMPS	E6/3UTR/A28G	–	G (5.4%/7.3%)	13	0	0.02	Creates miR23a binding site
2	rs3218592	REV3L	E/26/C8285T	R2762Q	T (1.8%/2.2%)	4	0	0.45	Predicted deleterious by Polyphen and SIFT
3	rs8071253	TK1	5UR//G-1181A	–	A (Unknown/3.4%)	6	0	0.26	Creates TGA1a binding site
4	rs501415	WDR7	I/1/A35G	–	G (2.3%/2.8%)	5	0	0.41	Disrupts a binding site of RUNX1 and possbily RUNX3 (Since all RUNX family proteins are sharing same motif)

SNP rs3218592 causes a non-conservative amino acid change in the REV3L gene which encodes the catalytic subunit of DNA Polymerase Zeta. DNA Polymerase Zeta was reported to be significantly down regulated in human colorectal cancer [34] and was suggested to be a tumour suppressor [35]. The T allele of the rs3218592 (E/26/C8285T, R2762Q) which is uniquely found in non-responder in our study is predicted to be damaging to the protein function by both Polyphen [36] and SIFT [37]. We thus hypothesize that the patients carrying this deleterious allele would have more aggressive disease and hence are more likely to be non-responsive to treatment.

pfSNP (rs8071253) resides in the promoter region of TK1 and is predicted to create a xenobiotic-stress activated TGA1a transcription binding site.

The third SNP (rs501415) resides within the first intron of WDR7 and is predicted to disrupt AML1 (RUNX) transcription factor bind site. Since the entire RUNX family share the same binding site (TGt/cGGT) [19], we postulate that this SNP may also affect the binding of RUNX3. RUNX3 had been hailed as a tumour suppressor gene and reduced expression of RUNX3 has been previously associated with poorer survival in colorectal cancer patients [38]. Nonetheless, the role of WDR7 in 5-FU resistance remained unclear. It was reported to be associated with 5-FU by PharmGKB [39] but the publication [39] was recently retracted [40].

### The logistic regression-based multivariate model identified an additional 5 pfSNPs which are not non-responder-specific that may distinguish responders from non-responders

**Figure 2:**
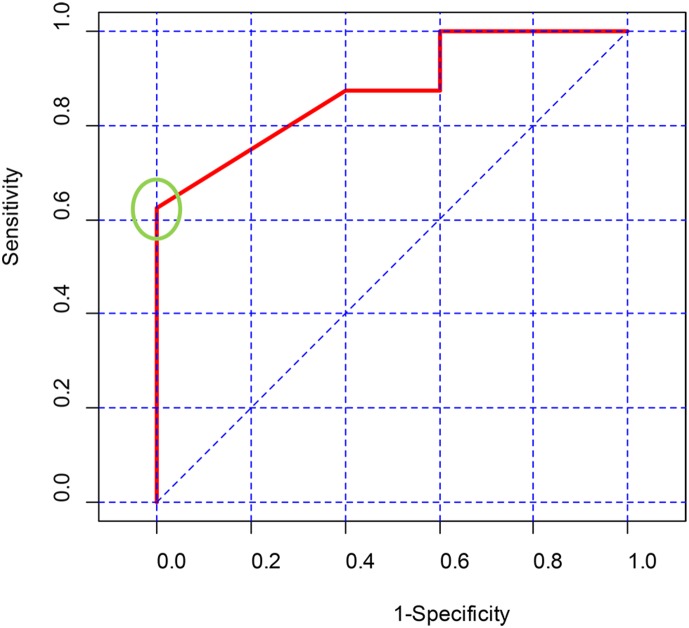
The ROC curve for the logistic regression based multivariate model trained to differentiate non-responders who do not have the non-responder. The AUC of the ROC curve is 0.875. The point of maximum sum of sensitivity and specificity is highlighted by the green circle on the ROC curve. The corresponding sensitivity and specificity is 62.5% and 100% respectively.

In addition to the non-responder specific pfSNPs that are associated with patients who do not respond to 5-FU, we proceeded to identify additional pfSNPs that may be associated with 5-FU drug response by training a logistic regression-based multivariate model with the stepAIC method using data from the other pfSNPs. The multivariate model identified 5 additional pfSNPs, namely, rs2289310 (DLG5, E/23/G4442T, P1481Q), rs1047840 (EXO1, E/12/G1765A, E589K), rs17431184 (PTEN, I/7/T-400C), rs2236722 (CYP19A1, E/2/A115G, W39R) and rs17160359 (ABCB1, 5UR//G-4254T) (**Table 5**) that may distinguish responders from non-responders. The AUC (Area Under Curve) for ROC (Receiver Operating Characteristic) curve of these 5 SNPs is 0.875 (**Figure 2**). A predicted value of 0.794 was identified as the optimal cut-off point to predict drug response, with sensitivity of 62.5% and specificity of 100%. With this threshold, the logistic-based multivariate model can correctly identify 39.1% (25/64) of non-responders. Together with the 37.5% of non-responders predicted by the non-responder-specific SNPs, a total of 76.6% (49/64) of non-responders can be correctly identified by both models.

**Table 5:** The list of common SNPs included into the multi-variate model and their predicted molecular functions.

S.N	rsNo	Gene Name	mRNALocation	AA Change	Allele	Allele Count (Frequency)		Potential Molecular Function	Discussion
						NR (128)	Rsp (50)		
1	rs2289310	DLG5	E/23/G4442T	P1481Q	T	22 (17%)	17 (34%)	Causes a non-conservedAA change in PDZ-likedomain. The PDZ-likedomain is important incell-junction according toSuperFamily database.	DLG5 is involved in maintaining the epithelial integrity. The T allele causes a non-conserved AA change in the PDZ-like domain which is important in cell-junction. The T allele has been associated with increased risk for Inflammatory Bowel Disease (IBD) and Crohn Disease (CD). The authors suggest it may have a role in altering tight-junction mediated permeability. It has been shown that enhanced intestinal permeability would increase 5-FU absorption. Therefore, patients with T allele may have higher 5-FU bioavailability and more likely to respond.
2	rs1047840	EXO1	E/12/G1765AE/13/G1765A	E589K E589K	A	20 (16%)	15 (30%)	Causes a non-conservedAA change in theExonuclease domain.	EXO1 is a gene controlling DNA repair (OMIM 606063). The A allele causes a non-conserved AA change in the exo-nuclease domain. The A allele has been associated with higher colorectal cancer risk in a UK population. We hypothesize that the increased risk is due to the reduced DNA repairing function of EXO1 caused by the A allele. Therefore, patients having A allele may be more responsive to DNA damaging agents like 5-FU.
3	rs17431184	PTEN	I/7/T-400C	–	C	15 (12%)	13 (26%)	Creates intronic splicingregulatory elements andunder recent positiveselection	PTEN is a well-known tumor suppressor and part of the apoptosis signalling pathway. The C allele is under recent positive selection. Therefore, patients having this allele may have stronger PTEN activity and respond better to 5-FU.
4	rs2236722	CYP19A1	E/2/A115GE/3/A115G	W39R W39R	G	16 (13%)	1 (2%)	Causes a non-conservedAA change in theCYP_P450 family domain.Predicted to be deleteriousby Polyphen. Also altersexonic splicing element.	A number of SNPs in the CYP19A1 gene have been associated with colorectal cancer risk. In the particular paper, this SNP has not been tested in this study because the study was conducted in an US white population and the SNP is likely to be mono-morphic according to HapMap. The G allele is detrimental to the protein function therefore patients having the G allele should have lower oestrogen level. This may translates to a higher colorectal cancer risk and more progressive disease therefore less responsive to 5-FU.
5	rs17160359	ABCB1	5UR/G-4254T	–	T	3 (2.3%)	5 (10%)	Creates binding sitefor HMGA1.	ABCB1 does not transport 5-FU but a few SNPs in it are implicated in CRC risk. The T allele creates a binding site of HMGA1 which is upregulated in various cancers.

It is noteworthy that the SNPs in the multivariate model are primarily localized within tumor suppressor gene (PTEN) or in genes which are mainly associated with colorectal cancer (DLG5, EXO1, CYP19A1 and ABCB1) (**Table 5**). Notably, one of the non-responder-specific SNP (rs3218592, REV3L E/26/C8285T, R2762Q) (**Table 4**) residing in the gene, Rev3L, has also been implicated to be a tumor suppressor gene [41].

The PTEN (phosphatase and tensin homolog) gene is a well-known tumor suppressor gene that was recently reported to control DNA repair and sensitivity to genotoxic stress [42]. The higher frequency of the C allele in the responders (26% in the responders vs. 12% in the non-responders) suggests that patients with this allele may exhibit lower tolerance to genotoxic stress caused by DNA damaging agents like 5-FU.

The DLG5 (Disks large homolog 5) gene encodes a member of the membrane-associated guanylate kinase (MAGUK) family of scaffolding proteins which is involved in maintaining the epithelial integrity [43]. The T allele of rs2289310 (DLG5, E/23/G4442T, P1481Q) causes a non-conserved amino acid change in the vicinity of one of the PDZ domains in this gene (aa 1391–1472; Prosite score 13.531). This variant was postulated to impair the scaffolding functions of DLG5 [44] and enhance the tight junction-mediated gut permeability [45]. This polymorphism has also been associated with increased risk of Inflammatory Bowel Disease [45] which may lead to increased risk for colorectal cancer [46]. Since enhanced gut permeability was reported to lead to higher 5-FU absorption in rats [47], we hypothesize that the T allele would lead to better drug absorption and thus better response. Consistent with our hypothesis, more responders have the T allele (MAF of 34% in responder vs 17% in non-responder) in this study.

The EXO1 (exonuclease 1) gene has been implicated to play roles in DNA replication, recombination, repair, telomere integrity [48] as well as damage signalling decisions [49]. The A allele of rs1047840 (EXO1, E/12/G1765A or E/13/G1765A, E589K) causes a non-conserved amino acid change and is predicted to reside within a region that is highly conserved amongst the XP-G/RAD2 DNA Repair Endonuclease Family (HMMPanther PTHR11081). In the Kin-cohort analyses, the A allele has been associated with higher risk of colorectal cancer in a UK population [50]. This SNP has also been associated with higher risk for various other cancers in the Chinese population [51]–[57]. We hypothesize that the increased cancer risk associated with this SNP could be due to less efficient repair of DNA damage in individuals carrying the A-allele. As the metabolites of 5-FU gets incorporated into DNA damaging the host DNA, the inefficient repair mechanisms of individuals carrying the A-allele of this SNP may result in greater cell death hence enhancing the effectiveness of 5-FU treatment. This is consistent with our observations that greater percentage of responders carries the A-allele of this SNP compared to non-responders (30% versus 16%).

CYP19A1 (cytochrome P450, family 19, subfamily A, polypeptide 1) or Aromatase is a member of the cytochrome P450 superfamily of enzymes and plays an important role in the metabolism of oestrogens. Oestrogen has been associated with lower risk of colorectal cancer [58]–[60]. Although several SNPs in the CYP19A1 gene had been reported to be associated with risk for colorectal cancer in a Caucasian population [61], SNP rs2236722 (E/2/A115G or E/3/A115G, W39R) was not examined in that study as it is monomorphic in HapMap CEU population. Nonetheless, this SNP (rs2236722), which occurs at a frequency of 3.3% in HapMap CHB population and 9.5% in our study, causes a non-conserved amino acid change from hydrophobic tryptophan to charged arginine in the CYP_P450 family domain and is predicted by Polyphen [36] to be a deleterious alteration. Hence, it is possible that this deleterious change in CYP19A1 may lead to lower oestrogen levels leading to higher colorectal cancer risk. We thus hypothesize that patients with the minor G-allele may have more progressive disease and hence are less responsive to 5-FU treatment.

The final gene implicated by the multivariate model to be associated with 5-FU response is the ABCB1 (ATP-Binding Cassette, Sub-Family B (MDR/TAP), Member 1) or the MDR1 (multidrug resistance protein 1). Although 5-FU is not a substrate of MDR1 protein, there’s some, albeit controversial evidence that SNPs within the ABCB1 gene, may be associated with CRC risk [62], [63]. Nonetheless, the SNP rs17160359 (ABCB1, 5UR/G-4254T) which is implicated in the multivariate model to be associated with drug response in CRC patient, resides in the promoter region and the T allele of the SNP creates a binding site of a transcription factor called HMGA1 which is expressed at very low level in adult human tissues but highly expressed in various tumours [64].

In summary, three perfect LD, non-responder-specific pfSNPs within the UMPS gene which plays a role in 5-FU metabolism together with 3 other non-responder-specific pfSNPs and 5 other pfSNPs in genes that may play roles in modulating tumor risks may collaborate to influence the patient’s response of CRC drugs. This study thus provides one of the building blocks for subsequent meta-analysis in larger cohort of patients.

## Supporting Information

Figure S1 **The different sequencing patterns generated by the different genotype of the VNTR and embedded SNP in the TYMS gene promoter region.**
(PDF)

Figure S2 **The gene and pathway distribution of pfSNPs chosen for genotyping.**
(JPG)

Figure S3 **The number of SNPs selected for genotyping in each gene region and function category.**
(PDF)

Figure S4 **Comparing HapMap CHB reported allele frequency (Release 27) and allele frequency observed in this study.**
(PDF)

Figure S5 **The MAF distribution of GoldenGate genotyped SNPs.**
(JPG)

Table S1 **The list of all the SNPs included on the GoldenGate array.**
(PDF)

Table S2 **The 14 markers not suitable to be genotyped by GoldenGate array and genotyped by other methods.**
(PDF)

Table S3 **The MAF for SNPs selected for genotyping in each region based on HapMap R27 CHB.**
(PDF)
