# Supplementary figures and images for: Potentially Functional SNPs (pfSNPs) as Novel Genomic Predictors of 5-FU Response in Metastatic Colorectal Cancer Patients
Source: PLoS One. 2014 Nov 5;9(11):e111694. doi: 10.1371/journal.pone.0111694 (PMC4221105; doi:10.1371/journal.pone.0111694)

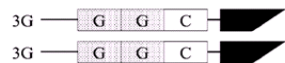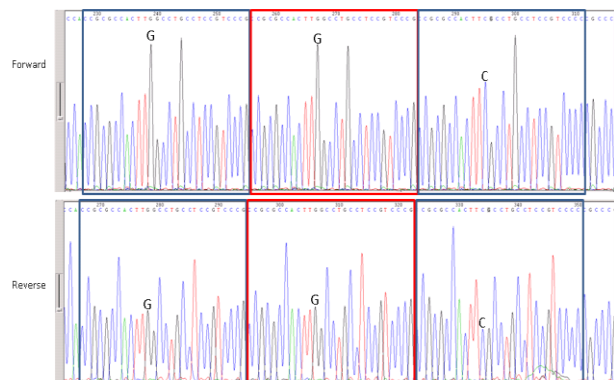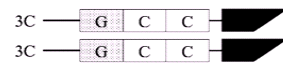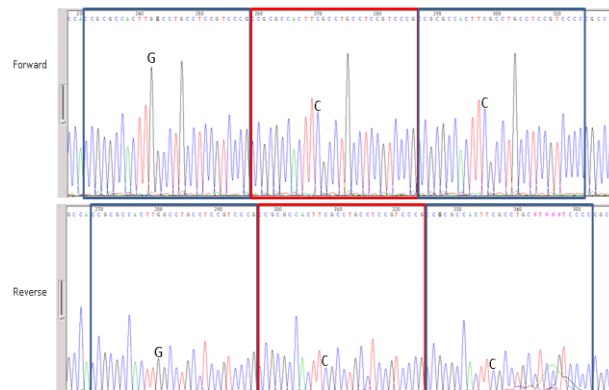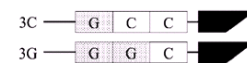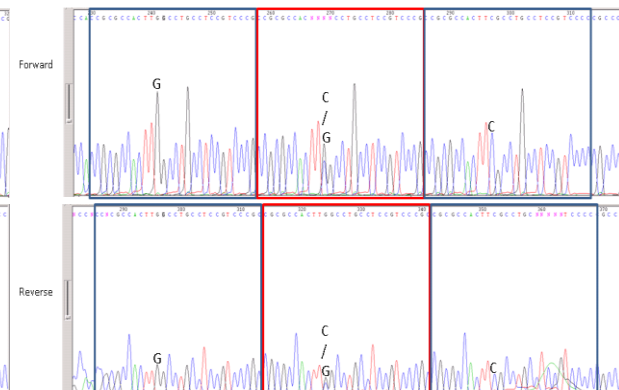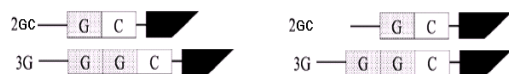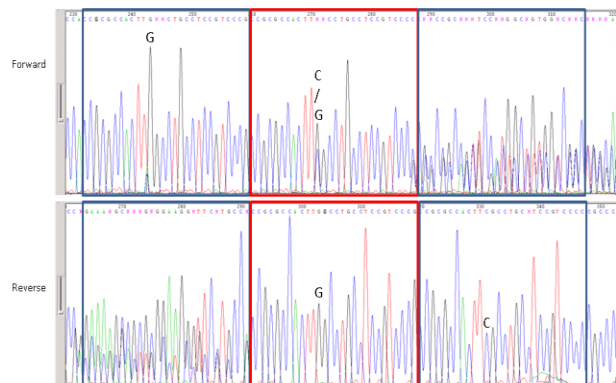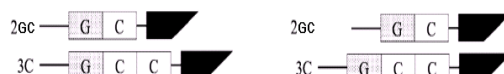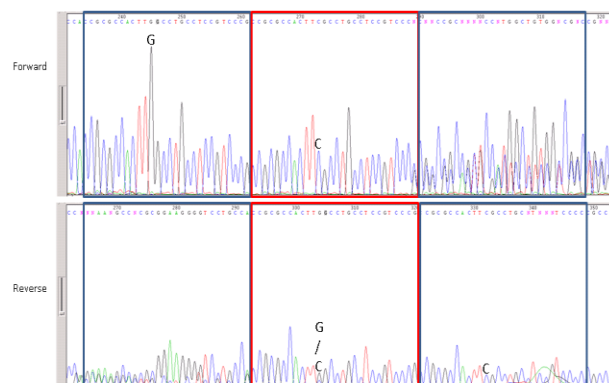

Supplement: Figure S1 — The different sequencing patterns generated by the different genotype of the VNTR and embedded SNP in the TYMS gene promoter region. (PDF) [file pone.0111694.s001.pdf]

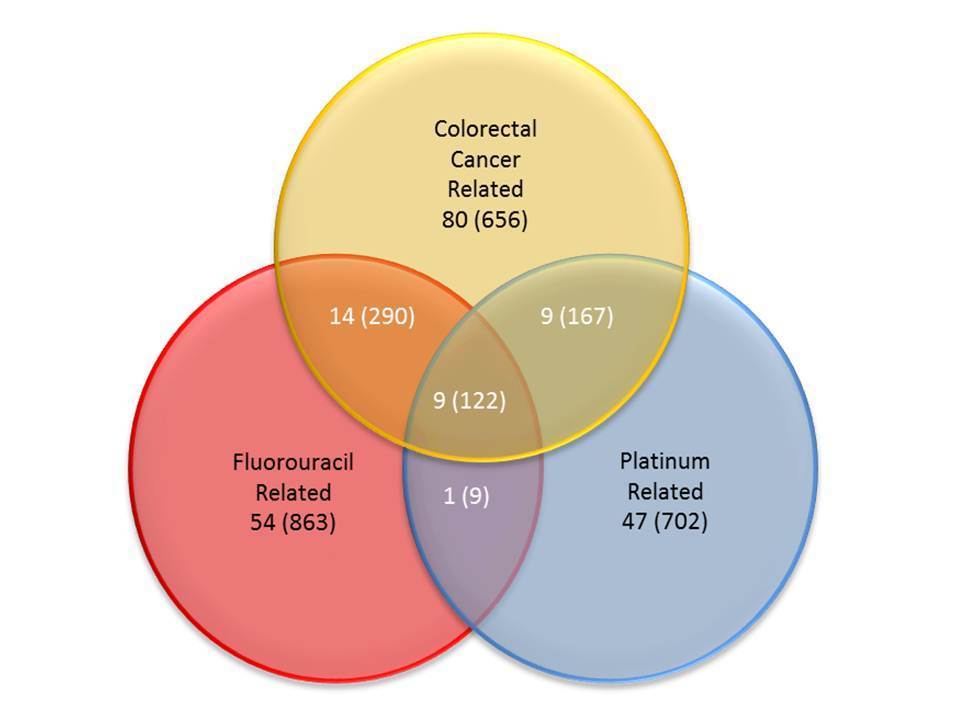

Supplement: Figure S2 — The gene and pathway distribution of pfSNPs chosen for genotyping. (JPG) [file pone.0111694.s002.jpg]

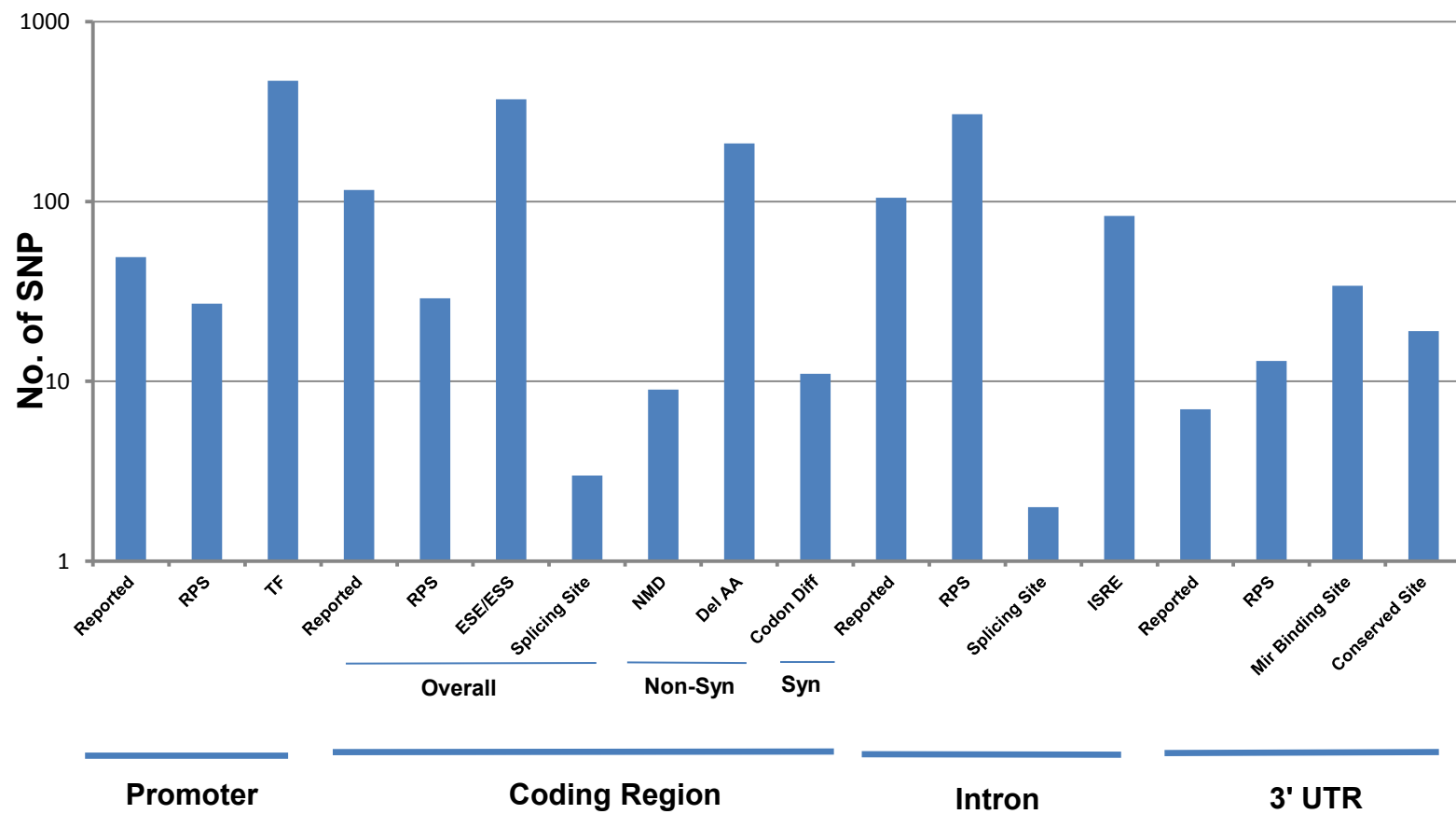

Supplement: Figure S3 — The number of SNPs selected for genotyping in each gene region and function category. (PDF) [file pone.0111694.s003.pdf]

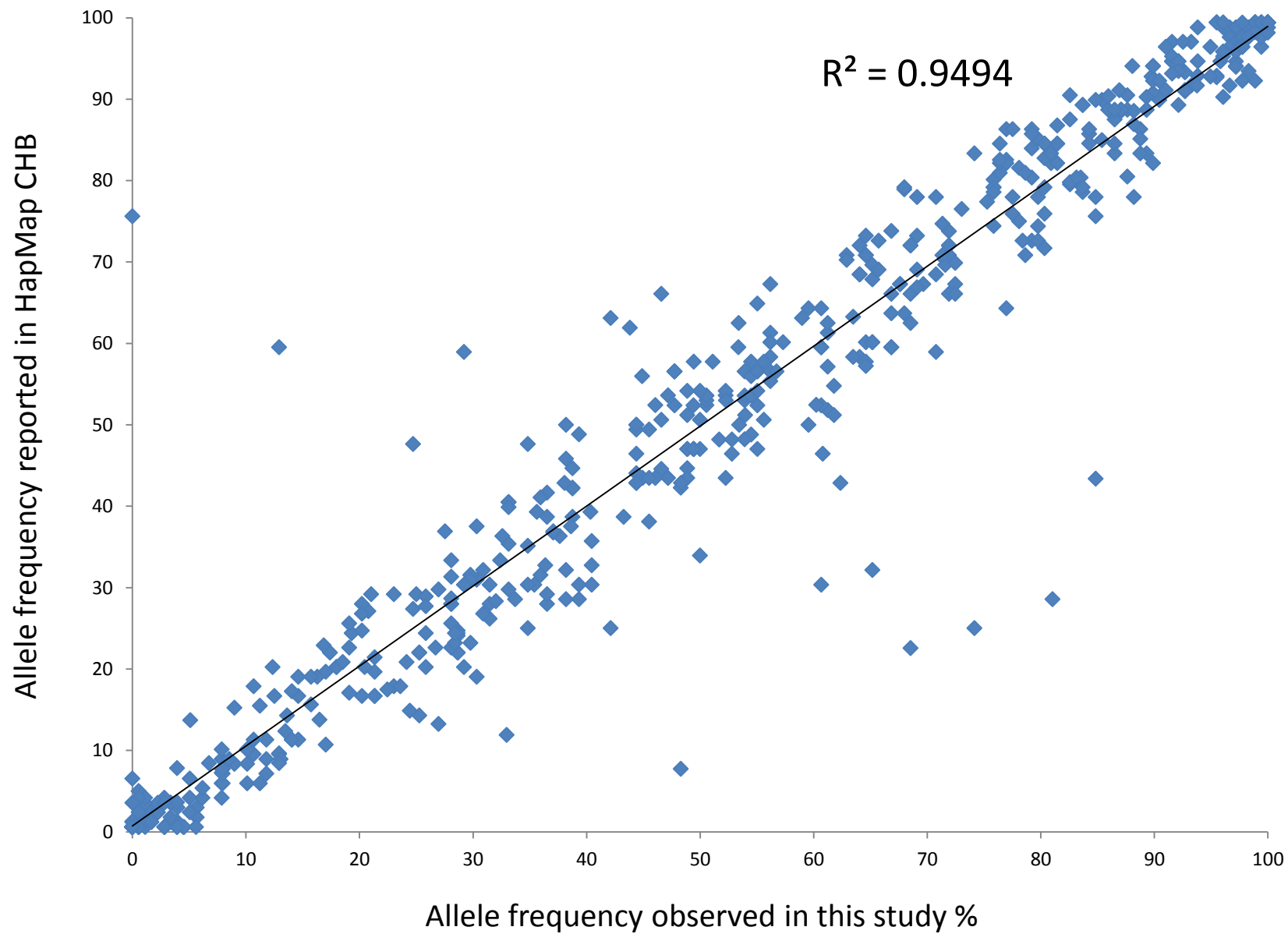

Supplement: Figure S4 — Comparing HapMap CHB reported allele frequency (Release 27) and allele frequency observed in this study. (PDF) [file pone.0111694.s004.pdf]

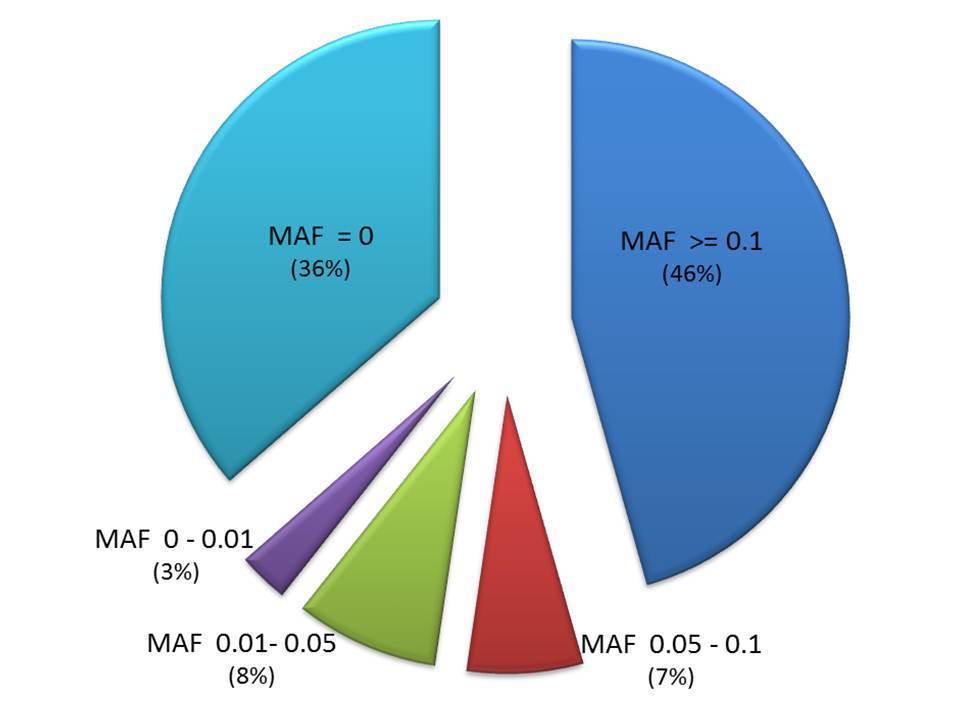

Supplement: Figure S5 — The MAF distribution of GoldenGate genotyped SNPs. (JPG) [file pone.0111694.s005.jpg]
