# Supplementary material for: Potentially Functional SNPs (pfSNPs) as Novel Genomic Predictors of 5-FU Response in Metastatic Colorectal Cancer Patients
Source: PLoS One. 2014 Nov 5;9(11):e111694. doi: 10.1371/journal.pone.0111694 (PMC4221105; doi:10.1371/journal.pone.0111694)
Supplement: Table S1 — The list of all the SNPs included on the GoldenGate array. (PDF) [file pone.0111694.s006.pdf]

Table S1. The list of all the SNPs included on the GoldenGate array.

#	rsNo	Gene	mRNA Location	AA Change
1	rs10046	CYP19A1	E10/3UTR /G19A	--
2	rs1010167	GSTM4	E/1/C-285G	--
3	rs1041983	NAT2	E/2/C282T	Y94Y
4	rs1042522	TP53	E/4/G215C	P72R
5	rs1042636	CASR	E/7/A2968G	R990G
6	rs1047840	EXO1	E/12/G1765A	E589K
7	rs1048572	SMARCE1	I/4/A1465G	--
8	rs10493895	DPYD	I/3/A22444C	--
9	rs10502975	DCC	I/20/C1225G	--
10	rs10508022	ABCC4	I/19/T11361C	--
11	rs1051266	SLC19A1	E/2/T80C	H27R
12	rs10513348	HLTF	I/23/G975A	--
13	rs10515959	DCC	I/15/G-19191T	--
14	rs1052555	ERCC2	E/22/G2133A	D711D
15	rs1052748	PLD2	E/17/C1730T	T577I
16	rs1056836	CYP1B1	E/3/C1294G	--
17	rs1057910	CYP2C9	E/7/A1075C	I359L
18	rs1059234	CDKN1A	E3/3UTR /C20T	--
19	rs10875053	DPYD	I/20/C21659G	--
20	rs10903118	RUNX3	5UR//T-3376C	--
21	rs10934683	UMPS	I/1/C-557T	--
22	rs10947623	CDKN1A	5UR//G-3650A	--

23	rs11202592	PTEN	E/1/C-9G	--
24	rs1143627	IL1B	5UR//G-30A	--
25	rs1145231	PMS1	E/9/T1181C	M394T
26	rs11545078	GGH	E/5/G452A	T151I
27	rs11549467	HIF1A	E/12/G1762A	A588T
28	rs11569017	EGF	E/15/A2351T	D784V
29	rs11615	ERCC1	E/3/A354G	N118N
30	rs11664579	WDR7	I/13/A323G	--
31	rs12052058	SMARCA4	I/30/G7289T	--
32	rs12052201	SMARCA4	I/30/G6860T	--
33	rs1208	NAT2	E/2/G803A	R268K
34	rs1234213	PTEN	I/3/G-1482A	--
35	rs12458289	BCL2	3DR//G6601T	--
36	rs12917	MGMT	E/5/C250T	L84F
37	rs12954274	DCC	I/14/C-209T	--
38	rs1314	UPB1	E10/3UTR /T690G	--
39	rs131794	TYMP	5UR//A-3296C	--
40	rs13181	ERCC2	E/23/T2251G	K751Q
41	rs1367634	DCC	I/14/T-1415G	--
42	rs1381547	BCL2	3DR//T27848C	--
43	rs1402001	ABCC5	3DR//A58790G	--
44	rs16260	CDH1	5UR//C-160A	--
45	rs1650697	DHFR	E/1/A-473G	--
46	rs1657396	WDR7	E27/3UTR /T1969C	--
47	rs1657415	WDR7	I/25/G19951A	--

48	rs16950632	ABCC4	I/20/G6290A	--
49	rs17189467	ABCC4	I/4/A-1931T	--
50	rs17217716	MSH2	E/1/C23T	T8M
51	rs17217772	MSH2	E/3/A380G	N127S
52	rs17224367	MSH2	E/7/C1168T	L390F
53	rs17630758	SMARCB1	I/3/G667A	--
54	rs17655	ERCC5	E/15/G3310C	D1104H
55	rs17756073	BCL2	3DR//A175404G	--
56	rs1787479	WDR7	I/25/C-24771G	--
57	rs1799782	XRCC1	E/6/G580A	R194W
58	rs1799794	XRCC3	E/2/T-315C	--
59	rs1799930	NAT2	E/2/G590A	R197Q
60	rs1800371	TP53	E/4/G139A	P47S
61	rs1800566	NQO1	E/5/G445A	P149S
62	rs1800734	MLH1	5UR//G-32A	--
63	rs1800975	XPA	E/1/T-4C	--
64	rs1801126	OGG1	E/1/G-18T	--
65	rs1801131	MTHFR	E/8/T1286G	E429A
66	rs1801133	MTHFR	E/5/G665A	A222V
67	rs1801158	DPYD	E/13/C1601T	S534N
68	rs1801166	APC	E/16/G3949C	E1317Q
69	rs1801265	DPYD	E/2/G85A	C29R
70	rs1801268	DPYD	E/23/C2983A	V995F
71	rs1801270	CDKN1A	E/2/C93A	S31R
72	rs1804197	APC	E16/3UTR /C86A	--

73	rs1810132	ERBB2	I/4/C-61T	--
74	rs1811086	TK1	I/4/G-1323A	--
75	rs1860460	PMS2	5UR//G-4548A	--
76	rs1922242	ABCB1	I/17/A-76T	--
77	rs1926657	ABCC4	I/4/T11908C	--
78	rs1950902	MTHFD1	E/6/A401G	R134K
79	rs1979277	SHMT1	E/11/G1303A	L435F
80	rs2020873	MLH1	E/19/C2152T	H718Y
81	rs2020911	MSH6	I/5/A14T	--
82	rs2020912	MSH6	E/4/T2633C	V878A
83	rs2066109	SMARCA2	I/23/T-1115C	--
84	rs2066518	SMARCAL1	E/5/G1129C	G377R
85	rs2072671	CDA	E/1/A79C	K27Q
86	rs2073390	SMARCB1	I/1/G-373A	--
87	rs2078486	TP53	I/1/G-3143A	--
88	rs2083020	WDR7	I/20/A20363G	--
89	rs2227306	IL8	I/1/C-204T	--
90	rs2227311	RB1	I/17/A31453G	--
91	rs2228001	XPC	E/16/G2818T	L940M
92	rs2228527	ERCC6	E/18/T3637C	R1213G
93	rs2229080	DCC	E/3/C601G	R201G
94	rs2229992	APC	E/12/T1458C	Y486Y
95	rs2229995	APC	E/16/G7504A	G2502S
96	rs2231142	ABCG2	E/5/G421T	Q141K
97	rs2233919	SMUG1	E/3/G7A	Q3*

98	rs2234978	FAS	E/6/T579C	T193T
99	rs2235035	ABCB1	I/14/G81A	--
100	rs2236722	CYP19A1	E/2/A115G	W39R
101	rs2243115	IL12A	5UR//T-348G	--
102	rs2244500	TYMS	I/2/A-1141G	--
103	rs2250889	MMP9	E/10/G1721C	R574P
104	rs2270951	DCC	I/21/C127T	--
105	rs2274976	MTHFR	E/12/C1781T	R594Q
106	rs2277448	ATP7B	E/1/G-75T	--
107	rs2278495	WDR7	I/25/G-163A	--
108	rs2279115	BCL2	5UR//G-223T	--
109	rs2289310	DLG5	E/23/G4442T	P1481Q
110	rs2299939	PTEN	I/2/C3284A	--
111	rs2302323	PLD2	E/25/G2702A	G901D
112	rs2303428	MSH2	I/12/T-6C	--
113	rs2308321	MGMT	E/7/A427G	S143P
114	rs2308327	MGMT	E/7/A533G	G178E
115	rs2355164	UPP2	I/2/C27144T	--
116	rs2376311	SMARCA2	I/25/G666A	--
117	rs238406	ERCC2	E/6/T468G	R156R
118	rs240993	REV3L	I/18/T-720C	--
119	rs243865	MMP2	5UR//C-1281T	--
120	rs2517954	ERBB2	5UR//T-12703C	--
121	rs2517955	ERBB2	5UR//C-12572T	--
122	rs2517956	ERBB2	5UR//G-12394A	--

123	rs25487	XRCC1	E/10/T1196C	R399Q
124	rs25489	XRCC1	E/9/C839T	R280H
125	rs25496	XRCC1	E/3/A215G	V72A
126	rs2660744	PPAT	E/11/G1462A	Q488*
127	rs2665797	SMARCD2	5UR//G-2133C	--
128	rs2735343	PTEN	I/5/G-6446C	--
129	rs28366003	MT2A	5UR//A-19G	--
130	rs28399504	CYP2C19	E/1/A1G	M1V
131	rs2853741	TYMS	5UR//T-298C	--
132	rs28756987	MLH3	E/2/G1939A	R647C
133	rs3092856	ATM	E/2/C94T	H32Y
134	rs3136367	MSH6	I/8/C-40G	--
135	rs3136788	POLB	I/11/A-2634G	--
136	rs3136797	POLB	E/12/C725G	P242R
137	rs3176734	XPA	I/5/G-2522A	--
138	rs3212931	ERCC1	5UR//G-137T	--
139	rs3218599	REV3L	E/13/C5434G	D1812H
140	rs3219090	PARP1	I/13/T118C	--
141	rs34035085	UPB1	E/2/C254A	A85E
142	rs34136999	MSH2	E/5/C815T	A272V
143	rs34213726	MLH1	E/12/A1327C	K443Q
144	rs34330	CDKN1B	E/1/T-79C	--
145	rs34374438	MSH6	E/4/A2561T	K854M
146	rs34986638	MSH2	E/14/G2422T	E808*
147	rs35001569	MLH1	E/16/A1852G	K618E

148	rs35032294	MLH1	5UR//C-208G	--
149	rs35045067	MLH1	E/17/A1937G	Y646C
150	rs351855	FGFR4	E/9/G1162A	G388R
151	rs35917308	PTEN	E/4/C234T	P78P
152	rs3731249	CDKN2A	E/2/C442T	A148T
153	rs3732183	MSH2	I/10/G12A	--
154	rs3738888	BARD1	E/10/G1972A	R658C
155	rs3740066	ABCC2	E/28/C3972T	I1324I
156	rs3744951	BCL2	3DR//T59207C	--
157	rs3749441	ABCC5	3DR//C40735G	--
158	rs3755319	UGT1A1	5UR//A-1336C	--
159	rs3758149	GGH	5UR//G-341A	--
160	rs3758581	CYP2C19	E/7/G991A	I331V
161	rs3764496	DCC	I/15/T-61C	--
162	rs3789243	ABCB1	I/4/A4196G	--
163	rs3790674	UCK2	I/5/A1103G	--
164	rs3792582	ABCC5	3DR//A6939G	--
165	rs3793784	ERCC6	5UR//G-466C	--
166	rs3794917	DCC	I/16/G-1683T	--
167	rs3794922	DCC	I/16/G1739A	--
168	rs3803185	ARL11	E/2/T442C	C148R
169	rs3805112	ABCC5	3DR//C55879T	--
170	rs3808607	CYP7A1	5UR//G-202T	--
171	rs3817672	TFRC	E/4/C424T	S142G
172	rs3918290	DPYD	I/14/C1T	--

173	rs4105144	CYP2A6	5UR//T-2283C	--
174	rs41294980	MLH1	E/12/G1217A	S406N
175	rs41295278	MSH6	E/9/A3961G	R1321G
176	rs4150001	EXO1	E/14/G2276A	G759E
177	rs4150521	ERCC3	E/14/G2111A	S704L
178	rs41549115	ERCC2	E/2/G72A	Y24Y
179	rs42427	APC	E/16/G5034A	G1678G
180	rs4246514	DPYD	I/2/C-3979G	--
181	rs4253038	ERCC6	I/3/A-408G	--
182	rs4253208	ERCC6	E/18/G3284C	P1095R
183	rs4372296	DPYD	I/2/C-26740A	--
184	rs4379706	DPYD	I/2/C26441T	--
185	rs4421623	DPYD	I/2/T-22533G	--
186	rs4516035	VDR	5UR//T-1011C	--
187	rs459552	APC	E/16/T5465A	V1822D
188	rs4645878	BAX	5UR//A-178G	--
189	rs465899	APC	E/16/G5880A	P1960P
190	rs4673	CYBA	E/4/A214G	Y72H
191	rs4773866	ABCC4	I/1/C24169T	--
192	rs4775936	CYP19A1	I/1/C-875T	--
193	rs4986783	NAT1	E/3/T640G	S214A
194	rs4986909	CYP3A4	E/11/G1247A	P416L
195	rs4986913	CYP3A4	E/12/G1399A	P467S
196	rs4987188	MSH2	E/6/G965A	G322D
197	rs527912	CDA	I/2/G2751A	--

198	rs532545	CDA	5UR//C-271T	--
199	rs56131651	ABCC2	E/7/-842-	--
200	rs6413420	CYP2E1	5UR//G-37T	--
201	rs6413438	CYP2C19	E/5/C680T	P227L
202	rs6566846	WDR7	I/21/T-2912G	--
203	rs6593634	DPYD	I/20/A23413C	--
204	rs6665429	DPYD	I/14/C-5602T	--
205	rs671	ALDH2	E/12/G1510A	E504K
206	rs6759948	UPP2	I/2/A17195G	--
207	rs689466	PTGS2	5UR//T-1194C	--
208	rs717620	ABCC2	E/1/C-24T	--
209	rs7234941	BCL2	3DR//C62336T	--
210	rs7336051	ABCC4	I/1/C-26424T	--
211	rs7439366	UGT2B7	E/2/T802C	H268Y
212	rs762551	CYP1A2	I/1/C-154A	--
213	rs7646621	ABCC5	3DR//G60676T	--
214	rs7993619	ABCC4	I/19/A698C	--
215	rs7996205	ABCC4	3DR//G66320A	--
216	rs8082807	DCC	I/25/A-2265C	--
217	rs8094838	WDR7	I/21/A532G	--
218	rs8187699	ABCC2	E/27/A3817G	T1273A
219	rs8187710	ABCC2	E/32/G4544A	C1515Y
220	rs861539	XRCC3	E/7/G722A	T241M
221	rs869224	DCC	I/23/A766G	--
222	rs886205	ALDH2	E/1/A-360G	--

223	rs887829	UGT1A1	5UR//C-348T	--
224	rs945881	DPYD	I/13/G-29513A	--
225	rs955850	DPYD	I/14/T-9744C	--
226	rs9651118	MTHFR	I/2/T724C	--
227	rs965943	DCC	I/14/A-8653G	--
228	rs9807370	DCC	I/21/G719A	--
229	rs9946253	WDR7	I/18/A-2783C	--
230	rs9950970	DCC	I/14/T-2275C	--
231	rs10041507	SLCO6A1	I/3/T220C	--
232	rs10041525	SLCO6A1	I/3/T142C	--
233	rs1005793	BCL2	3DR//A95027G	--
234	rs10062613	SLCO6A1	I/11/C702T	--
235	rs10073892	SLCO6A1	I/10/T-21C	--
236	rs10081796	SLC31A1	I/1/C16220G	--
237	rs1011019	ERCC3	I/9/A463G	--
238	rs10124350	SMARCA2	I/23/A-2552G	--
239	rs10162199	ABCC4	I/11/C878T	--
240	rs10186677	UPP2	E/7/A462C	A154A
241	rs10204779	UPP2	I/2/G7261A	--
242	rs10276036	ABCB1	I/10/C-44T	--
243	rs1042040	PPAT	E11/3UTR /C1220G	--
244	rs10503079	BCL2	3DR//C72821T	--
245	rs10505058	DPYS	I/7/A4947G	--
246	rs10513190	SLC31A1	I/1/C9965T	--
247	rs1062472	ATP7A	E23/3UTR /T1960C	--

248	rs10747486	DPYD	I/3/A39932G	--
249	rs10757122	SMARCA2	I/1/T-4348C	--
250	rs10757185	SMARCA2	I/26/G1836T	--
251	rs10757188	SMARCA2	I/26/T-939C	--
252	rs10757211	SMARCA2	I/27/T12883G	--
253	rs10783069	DPYD	I/10/G-6470C	--
254	rs10811481	SMARCA2	I/27/C203T	--
255	rs10811504	SMARCA2	I/27/T5807C	--
256	rs10811515	SMARCA2	I/27/T8841C	--
257	rs10875098	DPYD	I/10/T-2194C	--
258	rs10964466	SMARCA2	I/1/A-45G	--
259	rs10964500	SMARCA2	I/3/A1359C	--
260	rs10964921	SMARCA2	I/27/G8243A	--
261	rs10965088	SMARCA2	I/28/G-9017A	--
262	rs10981699	SLC31A1	I/1/C11943T	--
263	rs11097408	SMARCAD1	I/6/T3505C	--
264	rs11202600	PTEN	I/2/G-2437C	--
265	rs11202607	PTEN	E9/3UTR /C2185T	--
266	rs11241185	APC	I/4/G3971A	--
267	rs1125205	SMARCA2	I/4/A-2508G	--
268	rs1132776	ABCC5	3DR//A5139G	--
269	rs11579252	CDA	I/2/A2681G	--
270	rs11665624	WDR7	I/25/G-27842A	--
271	rs11714840	SMARCC1	I/10/G800C	--
272	rs11746217	SLCO6A1	I/4/A-9G	--

273	rs118202	REV3L	I/21/G-1558T	--
274	rs11864810	CES1	5UR//T-2652G	--
275	rs11872907	WDR7	I/13/C4188T	--
276	rs11876256	WDR7	I/14/T9751A	--
277	rs11877604	WDR7	I/12/C-2229T	--
278	rs11879293	SMARCA4	I/1/G760A	--
279	rs1202168	ABCB1	I/7/G139A	--
280	rs12045999	DPYD	I/14/C7624T	--
281	rs12121543	MTHFR	I/7/C-76A	--
282	rs1234224	PTEN	I/2/A-9974G	--
283	rs1234225	PTEN	I/2/C-11701T	--
284	rs12345640	SMARCA2	I/27/T13816C	--
285	rs12380390	SMARCA2	I/5/A-1218G	--
286	rs12467193	UPP2	5UR//C-3548T	--
287	rs12511433	SMARCAD1	I/6/G268A	--
288	rs12571445	ERCC6	I/5/A-8125G	--
289	rs12610607	SMARCA4	I/1/A-9803G	--
290	rs12634398	ABCC5	I/2/A-1234G	--
291	rs12677953	GGH	I/7/C-100A	--
292	rs12921111	ERCC4	5UR//G-4199A	--
293	rs13090196	HLTF	I/23/C902G	--
294	rs13190449	SLCO6A1	E/1/G80A	A27V
295	rs1333717	DPYD	I/3/G29092A	--
296	rs13425206	MSH2	I/6/G-2037T	--
297	rs1370216	WDR7	I/26/G736A	--

298	rs1383596	BCL2	3DR//C76325A	--
299	rs1437069	WDR7	I/25/T-14715C	--
300	rs1437135	NQO1	I/1/A2508G	--
301	rs1452057	SLCO6A1	I/6/A4781G	--
302	rs1476413	MTHFR	I/10/C35T	--
303	rs1520025	PPAT	I/1/G1024A	--
304	rs1542005	WDR7	I/20/G8005A	--
305	rs1562961	SLCO6A1	I/10/A-2493G	--
306	rs1617844	ABCC4	I/8/G651A	--
307	rs1657410	WDR7	I/25/A-20153G	--
308	rs1657421	WDR7	I/25/C29064T	--
309	rs16851444	UCK2	I/1/T10643G	--
310	rs16861365	HLTF	E/15/T1506C	V502V
311	rs16930959	SLC31A1	I/1/A-6936G	--
312	rs16937154	SMARCA2	I/9/T-3581A	--
313	rs17017854	SMARCA5	I/14/G865C	--
314	rs17070841	BCL2	3DR//T87993C	--
315	rs17070861	BCL2	3DR//T78707G	--
316	rs17086746	PPAT	E11/3UTR /C1519T	--
317	rs17086758	PPAT	I/1/T-6672C	--
318	rs17107001	PTEN	I/3/G1195T	--
319	rs17139614	ATP7A	E23/3UTR /G2241C	--
320	rs17139617	ATP7A	I/12/C-882A	--
321	rs17189481	ABCC4	I/4/C4542T	--
322	rs17300865	ABCC4	I/4/G4340A	--

323	rs17431184	PTEN	I/7/T-400C	--
324	rs17471125	DPYD	I/21/T-5102C	--
325	rs1751021	ABCC4	I/8/C-2406T	--
326	rs1751029	ABCC4	I/8/A728G	--
327	rs17757541	BCL2	3DR//C105501G	--
328	rs17775180	ERCC6	I/8/C2846A	--
329	rs17785248	SMARCC1	I/24/A3765G	--
330	rs1787462	WDR7	I/25/A-28222G	--
331	rs1787463	WDR7	I/25/A-28121G	--
332	rs1787468	WDR7	I/25/C-27977T	--
333	rs1787475	WDR7	E27/3UTR /A1061G	--
334	rs1800668	GPX1	E/1/G-46A	--
335	rs1807999	BCL2	3DR//C83015G	--
336	rs184026	SMARCAL1	I/14/C642T	--
337	rs1871446	CDC2	E6/3UTR/A30G	--
338	rs1886261	SMARCA2	I/28/A-7981G	--
339	rs1893806	BCL2	3DR//C223A	--
340	rs1901512	SLCO6A1	I/12/T517C	--
341	rs1901521	SLCO6A1	I/9/G1986T	--
342	rs1901522	SLCO6A1	I/9/T5068G	--
343	rs1914	APC	I/8/A-6748T	--
344	rs1931063	DPYD	I/3/C30060T	--
345	rs1962292	SMARCA2	I/24/A164G	--
346	rs1962293	SMARCA2	I/24/T327C	--
347	rs2001776	SMARCA2	I/26/G1290A	--

348	rs2002042	ABCC2	I/19/C-2133T	--
349	rs2065943	DPYD	I/8/T-10704C	--
350	rs2066462	MTHFR	E/7/G1056A	S352S
351	rs2139512	PPAT	I/1/G-2737C	--
352	rs2214102	ABCB1	E/3/T-1C	--
353	rs2233913	SLC31A1	5UR//T-651C	--
354	rs2235046	ABCB1	I/17/T73C	--
355	rs2235048	ABCB1	I/27/G80A	--
356	rs2235074	ABCB1	I/4/G36A	--
357	rs2270860	SLC22A7	E/7/C1269T	S423S
358	rs2271937	ABCC5	3DR//G18581A	--
359	rs2273697	ABCC2	E/10/G1249A	V417I
360	rs2280392	ABCC5	3DR//A35799G	--
361	rs2281793	ERCC6	I/5/C4537T	--
362	rs2281794	ERCC6	I/5/T3659C	--
363	rs2282011	UCK1	I/2/G-444A	--
364	rs2293001	ABCC5	3DR//C1330T	--
365	rs2299941	PTEN	I/5/A-7156G	--
366	rs2306802	SMARCAD1	E/10/A1479G	Q493Q
367	rs232054	RRM1	I/2/C116G	--
368	rs2343614	REV3L	I/9/G-2511A	--
369	rs240954	REV3L	I/21/G-3009A	--
370	rs240955	REV3L	I/21/G-3830A	--
371	rs240991	REV3L	I/20/C2247G	--
372	rs240995	REV3L	I/18/T1704C	--

373	rs2431238	APC	I/6/T-3774C	--
374	rs2464803	APC	I/7/A3561G	--
375	rs2464805	APC	I/2/C-230A	--
376	rs2520464	ABCB1	I/5/C-1547T	--
377	rs2545162	APC	I/11/G743A	--
378	rs2546106	APC	I/8/C-5931A	--
379	rs2546108	APC	I/11/C-1336A	--
380	rs2546110	APC	I/11/A-392G	--
381	rs2576415	WDR7	I/19/T-30795A	--
382	rs2622604	ABCG2	I/1/T614C	--
383	rs2661681	TK1	I/5/C-4T	--
384	rs2707763	APC	I/4/T-2461C	--
385	rs2723877	TDG	I/1/T-1570C	--
386	rs2727280	SMARCD2	I/1/A1737G	--
387	rs2735691	RRM1	I/1/C1448G	--
388	rs2786498	DPYD	I/8/G-6425A	--
389	rs284564	SMARCAL1	I/4/T1088A	--
390	rs2848968	WDR7	I/25/G-20982A	--
391	rs2854510	XRCC1	I/2/A4850G	--
392	rs2909787	APC	I/14/G-2518C	--
393	rs2952615	APC	I/8/G1808C	--
394	rs3136748	POLB	I/5/C1214T	--
395	rs3176658	XPA	I/2/A-1942G	--
396	rs3176750	XPA	E/6/G754C	L252V
397	rs3212106	XRCC3	I/6/T-292C	--

398	rs3212121	XRCC3	E10/3UTR /T613C	--
399	rs3213255	XRCC1	I/2/G1555A	--
400	rs3213356	XRCC1	I/4/C-404T	--
401	rs351771	APC	E/14/G1635A	A545A
402	rs3740065	ABCC2	I/29/A154G	--
403	rs3745041	WDR7	I/13/C-199T	--
404	rs3749443	ABCC5	3DR//C62218T	--
405	rs3750417	SMARCA2	I/5/C-188T	--
406	rs3750748	ERCC6	I/5/C2665T	--
407	rs3750749	ERCC6	I/12/A33G	--
408	rs3758395	ABCC2	I/26/T154C	--
409	rs3774341	MLH1	I/3/A-659C	--
410	rs3780128	GGH	I/3/T-1214C	--
411	rs3782964	ABCC4	I/4/C-430T	--
412	rs3785911	ABCC3	I/30/A-1022C	--
413	rs3786362	TYMS	E/3/A381G	I127I
414	rs3789244	ABCB1	I/10/G1228T	--
415	rs3790661	UCK2	I/4/T2927C	--
416	rs3792584	ABCC5	3DR//T17631C	--
417	rs3793499	SMARCA2	I/27/C8053G	--
418	rs3805109	ABCC5	3DR//G11276A	--
419	rs3808830	UCK1	5UR//C-1778T	--
420	rs3810915	SLC31A1	5UR//A-2096G	--
421	rs3810944	ERCC6	I/19/A236G	--
422	rs3817403	ABCC5	3DR//A18396G	--

423	rs3829070	SMARCA2	I/26/T-80C	--
424	rs3829072	SMARCA2	I/27/G3859A	--
425	rs3829073	SMARCA2	I/27/T3946C	--
426	rs3829300	TDG	I/2/A12G	--
427	rs390092	APC	I/8/T-4995G	--
428	rs4135082	TDG	I/1/T-1409C	--
429	rs4135087	TDG	I/2/C387T	--
430	rs4135094	TDG	I/2/T-147C	--
431	rs4148328	UGT1A1	I/4/C574T	--
432	rs4148432	ABCC4	I/1/C-13075T	--
433	rs4148469	ABCC4	I/4/A-323G	--
434	rs4148485	ABCC4	I/9/G-2464A	--
435	rs4148568	ABCC5	I/2/C9031T	--
436	rs4148575	ABCC5	E7/3UTR /A447G	--
437	rs4148580	ABCC5	3DR//T16358C	--
438	rs4148584	ABCC5	3DR//A24040C	--
439	rs4148594	ABCC5	3DR//T61157C	--
440	rs4148738	ABCB1	I/21/C-2236T	--
441	rs4150402	ERCC3	I/3/T52C	--
442	rs4150456	ERCC3	E/9/C1485T	E495E
443	rs4150471	ERCC3	I/10/A2210G	--
444	rs4253055	ERCC6	I/5/A3752G	--
445	rs4253060	ERCC6	I/5/C4292T	--
446	rs4253095	ERCC6	I/5/A-1137G	--
447	rs4253101	ERCC6	I/6/T871G	--

448	rs4253121	ERCC6	I/7/G1597A	--
449	rs4253126	ERCC6	I/7/G-892T	--
450	rs4253138	ERCC6	I/8/C828T	--
451	rs4253165	ERCC6	I/9/C-128T	--
452	rs4253166	ERCC6	I/10/T716G	--
453	rs4303338	ABCC4	I/4/C10763G	--
454	rs4373572	SMARCA2	I/27/A632G	--
455	rs4537601	DPYD	I/3/G-27495C	--
456	rs453776	REV3L	I/21/T1665C	--
457	rs454886	APC	I/8/A-5075G	--
458	rs455645	REV3L	E/13/C4485T	S1495S
459	rs455650	REV3L	I/13/C2276T	--
460	rs460594	REV3L	I/20/C616G	--
461	rs461646	REV3L	E/13/G3301A	L1101L
462	rs4641140	SLC31A1	I/1/A1629G	--
463	rs4664236	UPP2	I/2/T17318G	--
464	rs4664920	UPP2	I/2/G7182A	--
465	rs4741637	SMARCA2	I/5/C3248G	--
466	rs4741638	SMARCA2	I/5/A3360T	--
467	rs4741640	SMARCA2	I/6/A-780C	--
468	rs4773854	ABCC4	I/4/G7454A	--
469	rs4940574	BCL2	3DR//G147343C	--
470	rs4941189	BCL2	3DR//C77260T	--
471	rs4970728	DPYD	I/4/G3787C	--
472	rs4987770	BCL2	3DR//C80832T	--

473	rs4987802	BCL2	3DR//C140464G	--
474	rs562	ABCC5	3DR//T63696C	--
475	rs614080	GSTP1	5UR//G-3998A	--
476	rs628959	DPYD	I/18/C31955T	--
477	rs6437129	UPP2	I/2/A-7469G	--
478	rs6475506	SMARCA2	I/27/G2301A	--
479	rs6475507	SMARCA2	I/27/A3532T	--
480	rs6475511	SMARCA2	I/27/T4991G	--
481	rs6475514	SMARCA2	I/27/G5170C	--
482	rs6475520	SMARCA2	I/27/A6891G	--
483	rs6475524	SMARCA2	I/27/A7597G	--
484	rs6567334	BCL2	3DR//A67807C	--
485	rs6683957	DPYD	I/4/A5229G	--
486	rs6768699	SMARCC1	I/8/A-450G	--
487	rs6790814	ABCC5	3DR//C44735G	--
488	rs6806313	ABCC5	3DR//G59389C	--
489	rs6854326	PPAT	I/1/T-2027C	--
490	rs6873738	SLCO6A1	I/12/A7020C	--
491	rs6877722	SLCO6A1	I/12/G-738T	--
492	rs6884141	SLCO6A1	E/2/G393A	G131G
493	rs6915753	REV3L	I/1/T-4004C	--
494	rs6922226	REV3L	I/4/A2189G	--
495	rs6935759	REV3L	I/1/G25233A	--
496	rs6942129	REV3L	I/1/T-25945C	--
497	rs6949448	ABCB1	I/26/T2733C	--

498	rs6961665	ABCB1	I/10/C-1264A	--
499	rs699937	POLH	5UR//G-3018A	--
500	rs699937	XPO5	I/2/G291A	--
501	rs7021817	SMARCA2	I/27/A16224G	--
502	rs7033529	SMARCA2	I/27/A1164G	--
503	rs7035071	SMARCA2	I/14/T1474C	--
504	rs7035608	SMARCA2	I/9/C3521T	--
505	rs7035898	SMARCA2	I/9/C3809T	--
506	rs7040174	SMARCA2	I/24/A-1116C	--
507	rs7040968	SMARCA2	I/27/G-12720A	--
508	rs7048496	SMARCA2	I/27/C1294A	--
509	rs7048976	SMARCA2	I/28/G-8275T	--
510	rs7073830	ERCC6	I/7/G-1932A	--
511	rs719717	ABCC3	I/1/C-3695G	--
512	rs720159	UPP2	5UR//G-2886A	--
513	rs7243516	WDR7	I/12/C-7239T	--
514	rs726511	BCL2	3DR//A72295T	--
515	rs731420	XRCC1	I/4/G-137A	--
516	rs748766	MLH1	I/14/T-885C	--
517	rs7506986	WDR7	I/14/G5730A	--
518	rs7618758	SMARCC1	I/6/C-258T	--
519	rs7654543	PPAT	I/1/A-8415C	--
520	rs7751272	REV3L	I/1/A-2596G	--
521	rs7764543	REV3L	I/1/T-6644C	--
522	rs7766610	REV3L	I/9/C1147A	--

523	rs7847382	SMARCA2	I/27/T4325G	--
524	rs7851702	SMARCA2	I/23/A-2352G	--
525	rs7853086	SMARCA2	I/26/A-299T	--
526	rs7858597	SMARCA2	I/27/C4461G	--
527	rs7862805	SMARCA2	I/27/T13414A	--
528	rs7864080	SMARCA2	I/11/A-52T	--
529	rs7864452	SLC31A1	I/1/C14630T	--
530	rs7869436	SMARCA2	I/27/A3378G	--
531	rs7920256	ERCC6	I/7/A-2848G	--
532	rs7927381	GSTP1	5UR//T-4542C	--
533	rs8001444	ABCC4	I/1/T1058C	--
534	rs8060569	MT1A	5UR//T-1437C	--
535	rs8073898	MPO	E12/3UTR /A561T	--
536	rs8086404	BCL2	3DR//G68063C	--
537	rs8093133	WDR7	I/12/T-444G	--
538	rs8096380	BCL2	3DR//G64706A	--
539	rs8187692	ABCC2	E/25/G3542T	R1181L
540	rs8192729	CYP2A6	I/7/C203T	--
541	rs861531	XRCC3	I/5/C533A	--
542	rs869951	ABCC4	5UR//C-522G	--
543	rs899497	ABCC4	I/4/A-108G	--
544	rs9400476	REV3L	I/1/C19578T	--
545	rs9472084	POLH	I/3/A-1658G	--
546	rs9487639	REV3L	I/1/G-10319A	--
547	rs9487645	REV3L	I/1/T-26593C	--

548	rs9524856	ABCC4	I/4/T588C	--
549	rs9524873	ABCC4	I/1/G26109A	--
550	rs9524885	ABCC4	I/1/T17906C	--
551	rs9561811	ABCC4	I/4/C3193T	--
552	rs956275	PPAT	I/7/C90T	--
553	rs9568682	ATP7B	I/1/C-15456G	--
554	rs9590228	ABCC4	I/1/C-23383T	--
555	rs9634642	ABCC4	I/3/T-1583C	--
556	rs971667	ERCC6	I/7/G3490A	--
557	rs9812777	ABCC5	3DR//C38368T	--
558	rs981988	SLCO6A1	I/1/T-3843C	--
559	rs9864549	HLTF	I/18/A-1738C	--
560	rs991791	WDR7	I/25/T-22204C	--
561	rs9936741	MT1A	5UR//T-4792C	--
562	rs9972996	BCL2	3DR//A63297G	--
563	rs2695784	SMARCC2	5UR//T-3166C	--
564	rs291592	DPYD	E23/3UTR /C768T	--
565	rs2930788	SMARCC2	5UR//T-2971C	--
566	rs3136717	POLB	I/1/C-89T	--
567	rs4801043	WDR7	5UR//T-1512C	--
568	rs2071487	GSTM1	E/7/T462C	T154T
569	rs4846049	MTHFR	E12/3UTR /T372G	--
570	rs733590	CDKN1A	5UR//T-1283C	--
571	rs10075210	ATOX1	5UR//G-771A	--
572	rs1008767	MT2A	5UR//T-2567C	--

573	rs10118903	FPGS	5UR//C-106T	--
574	rs10120113	SMARCA2	5UR//A-2574G	--
575	rs10121175	SMARCA2	5UR//G-4580A	--
576	rs1023159	SLC19A1	5UR//G-3828A	--
577	rs10239964	POLM	5UR//A-2978G	--
578	rs10252226	ABCB1	5UR//A-4823C	--
579	rs10261685	ABCB1	5UR//A-1853C	--
580	rs10265836	POLM	5UR//C-2395A	--
581	rs10269664	POLM	5UR//C-3062T	--
582	rs10424731	XRCC1	5UR//T-2582C	--
583	rs10458900	RRM1	5UR//C-4328G	--
584	rs1046512	MLH1	5UR//A-2682C	--
585	rs1057985	SLC29A1	5UR//T-1340C	--
586	rs10857503	ERCC6	5UR//C-2667T	--
587	rs10857795	GSTM1	I/1/G-25A	--
588	rs10929302	UGT1A1	5UR//G-3136A	--
589	rs10947622	CDKN1A	5UR//C-4084T	--
590	rs10951974	PMS2	5UR//C-2630T	--
591	rs10987740	FPGS	5UR//G-2689A	--
592	rs11101152	ERCC6	5UR//C-1488A	--
593	rs11111858	TDG	5UR//T-1934A	--
594	rs11133442	PPAT	5UR//A-4621T	--
595	rs1114357	SLC22A7	5UR//C-3068T	--
596	rs1130609	RRM2	E/1/T-6G	--
597	rs11400410	MSH6	5UR//--3687G	--

598	rs11445091	UMPS	5UR//--3982T	--
599	rs11594945	ERCC6	5UR//T-4153C	--
600	rs11649492	ERCC4	5UR//G-4604C	--
601	rs11653440	SMARCD2	5UR//T-2821C	--
602	rs11657432	TK1	5UR//C-3010A	--
603	rs11665663	FXYD3	5UR//T-4460C	--
604	rs11671498	XRCC1	5UR//G-4956C	--
605	rs11673726	UGT1A1	5UR//G-4858T	--
606	rs11681986	RRM2	5UR//G-1507A	--
607	rs11705654	GSTT1	5UR//A-2542G	--
608	rs11769882	POLM	5UR//G-2000A	--
609	rs11779233	DPYS	5UR//T-4702A	--
610	rs11790511	SLC31A1	5UR//C-3396T	--
611	rs11809445	GPX7	E/1/G-27T	--
612	rs11816547	ABCC2	5UR//T-3037C	--
613	rs11846751	XRCC3	5UR//C-2833G	--
614	rs11846751	ZFYVE21	I/1/C2341G	--
615	rs11846838	XRCC3	5UR//G-2913A	--
616	rs11846838	ZFYVE21	I/1/G2421A	--
617	rs11848956	XRCC3	5UR//G-4444T	--
618	rs11848956	ZFYVE21	I/1/G3952T	--
619	rs11865517	MT2A	5UR//G-1835A	--
620	rs11878644	ERCC2	5UR//T-3137C	--
621	rs11880627	ERCC1	5UR//G-3793A	--
622	rs11909852	SOD1	5UR//C-4004T	--

623	rs11933670	SMARCA5	E/1/G-183A	--
624	rs11948127	FGFR4	5UR//G-1395A	--
625	rs11971012	UPP1	5UR//T-3523C	--
626	rs12068997	GSTM1	E/2/C81T	S27S
627	rs12192827	CDKN1A	5UR//C-3515T	--
628	rs12199346	CDKN1A	5UR//C-4940A	--
629	rs12214686	CDKN1A	5UR//A-3935G	--
630	rs12379987	FPGS	5UR//T-3465C	--
631	rs12445357	CES1	5UR//T-1752C	--
632	rs12463451	ERCC3	5UR//C-2130T	--
633	rs12471171	ERCC3	5UR//G-1851A	--
634	rs12492095	UMPS	5UR//T-1256A	--
635	rs12526616	POLH	I/1/A16C	--
636	rs12526616	XPO5	5UR//A-528C	--
637	rs12536587	POLM	5UR//G-709C	--
638	rs12597222	MT2A	5UR//T-2922C	--
639	rs12621805	SMARCAL1	5UR//T-3123C	--
640	rs12632590	UMPS	I/1/C30T	--
641	rs12659155	ATOX1	5UR//C-1641T	--
642	rs12806698	RRM1	E/1/C-269A	--
643	rs12949848	MPO	5UR//A-3770G	--
644	rs12978764	FXYD3	5UR//C-4585T	--
645	rs13079924	SMARCC1	5UR//A-1323C	--
646	rs1319052	ERCC1	5UR//G-4043A	--
647	rs13206175	CDKN1A	5UR//A-1452T	--

648	rs13306560	MTHFR	5UR//C-67T	--
649	rs1331700	HMGB1	5UR//C-1834T	--
650	rs13330389	CES1	5UR//A-662G	--
651	rs13401024	UPP2	5UR//C-2098T	--
652	rs13405649	SMARCAL1	5UR//G-4805T	--
653	rs13414112	UPP2	I/1/G32C	--
654	rs1364362	ERCC4	5UR//T-3128C	--
655	rs1382539	DHFR	5UR//G-1353A	--
656	rs1382541	DHFR	5UR//T-2683C	--
657	rs1382542	DHFR	5UR//T-2780C	--
658	rs140313	GSTT1	I/1/C166T	--
659	rs140315	GSTT1	5UR//G-3437A	--
660	rs140316	GSTT1	5UR//C-4042T	--
661	rs140317	GSTT1	5UR//A-4410G	--
662	rs1465952	RRM1	5UR//G-1384A	--
663	rs1469908	NQO1	5UR//C-3878T	--
664	rs1473418	BCL2	E/1/C-428G	--
665	rs1510841	SMARCAL1	I/1/G115A	--
666	rs1520195	ABCC5	5UR//G-1154A	--
667	rs1526603	SMARCE1	5UR//C-1164T	--
668	rs1544105	FPGS	5UR//C-2428T	--
669	rs1549920	ATOX1	5UR//A-2633T	--
670	rs1554494	UPP1	5UR//G-165A	--
671	rs1561876	RRM1	5UR//G-2528A	--
672	rs1611028	DHFR	5UR//TT-2880-	--

673	rs1634252	GSTM1	5UR//T-1751C	--
674	rs1643639	DHFR	5UR//T-1589C	--
675	rs1662162	RRM1	5UR//T-659C	--
676	rs1677667	DHFR	5UR//C-1740G	--
677	rs16835902	UMPS	5UR//C-3448G	--
678	rs16835912	UMPS	5UR//C-3308A	--
679	rs16856038	SMARCAL1	5UR//C-3302G	--
680	rs1688038	FXYD3	5UR//G-1799C	--
681	rs1688039	FXYD3	5UR//A-1575T	--
682	rs16896398	SLC22A7	5UR//A-3293T	--
683	rs16929410	RRM1	5UR//G-382A	--
684	rs17037425	MTHFR	5UR//G-4267A	--
685	rs17160359	ABCB1	5UR//G-4254T	--
686	rs172814	TDG	5UR//T-1802C	--
687	rs1735068	RRM1	5UR//T-265G	--
688	rs17510346	REV3L	5UR//G-1937A	--
689	rs17511525	REV3L	E/31/G9045T	I3015I
690	rs1764416	ABCC4	5UR//T-3360C	--
691	rs1766902	ABCC4	5UR//T-3275C	--
692	rs17767961	CES2	5UR//G-4155A	--
693	rs17779585	SMUG1	5UR//C-2175G	--
694	rs17843768	UMPS	5UR//C-827A	--
695	rs17880282	TP53	5UR//C-1079T	--
696	rs17882503	SOD1	5UR//T-649C	--
697	rs17883184	TP53	5UR//G-636A	--

698	rs17883908	TP53	I/1/A236G	--
699	rs17884410	TP53	E/1/T-110C	--
700	rs17886079	TP53	5UR//C-342T	--
701	rs1799797	ERCC4	5UR//T-29A	--
702	rs1811322	MT2A	5UR//A-2805T	--
703	rs1827211	MT1A	5UR//C-4321T	--
704	rs1827212	MT1A	5UR//T-2674C	--
705	rs1827213	MT1A	5UR//G-736A	--
706	rs184239	XRCC1	5UR//G-4439A	--
707	rs1862849	MT2A	5UR//G-3016A	--
708	rs1863332	MSH2	5UR//T-364G	--
709	rs1863333	MSH2	5UR//T-658C	--
710	rs1917800	ERCC6	5UR//G-3844C	--
711	rs1944423	BCL2	5UR//A-3904G	--
712	rs1977172	CDKN1A	5UR//A-4732C	--
713	rs2009115	PMS2	5UR//G-3818A	--
714	rs2014704	SMARCE1	5UR//T-4145G	--
715	rs2020872	MLH1	E/1/A94G	I32V
716	rs2066461	MTHFR	E/3/G345T	T115T
717	rs2066466	MTHFR	E/3/C417T	T139T
718	rs2070473	UPB1	5UR//G-356A	--
719	rs2070474	UPB1	E/1/C-80G	--
720	rs2073387	SMARCB1	5UR//T-144G	--
721	rs2107545	MPO	5UR//A-1821G	--
722	rs2108811	UPP2	5UR//A-3048G	--

723	rs2144078	XRCC3	5UR//T-2523C	--
724	rs2144078	ZFYVE21	I/1/T2031C	--
725	rs2145851	ABCC2	5UR//C-4676A	--
726	rs2161737	MT2A	5UR//A-3276G	--
727	rs2180989	ABCC2	5UR//T-4761G	--
728	rs219240	SMARCD3	5UR//C-32793T	--
729	rs2231135	ABCG2	E/1/A-475G	--
730	rs2232861	UPB1	5UR//G-96A	--
731	rs2233914	SLC31A1	5UR//G-327A	--
732	rs2266635	GSTT1	E/1/C61T	A21T
733	rs2270836	MT1A	5UR//C-4963T	--
734	rs2276665	SMARCAL1	5UR//T-1005G	--
735	rs2276910	SMARCAD1	I/1/T17C	--
736	rs2287497	TP53	5UR//G-1862A	--
737	rs2287498	TP53	5UR//C-1642T	--
738	rs2287499	TP53	5UR//C-1250G	--
739	rs2297393	SLC29A1	5UR//T-2522C	--
740	rs2298840	DPYS	E/1/G216A	F72F
741	rs2307160	POLB	E/1/A-62G	--
742	rs2313211	ABCC5	5UR//T-2898A	--
743	rs2334102	TYMP	5UR//G-1296C	--
744	rs2395655	CDKN1A	5UR//A-790G	--
745	rs2429247	SMARCD3	5UR//T-28911C	--
746	rs244711	FGFR4	5UR//C-4727T	--
747	rs2448341	CDC2	5UR//C-3953T	--

748	rs2448342	CDC2	5UR//A-3883G	--
749	rs2463365	SMARCC2	5UR//C-4298T	--
750	rs2467573	TK1	5UR//A-286T	--
751	rs2517953	ERBB2	5UR//G-15042C	--
752	rs2546095	FGFR4	5UR//T-4336G	--
753	rs2584618	SMARCD2	5UR//T-3259C	--
754	rs2584879	RRM1	E/1/G17A	R6Q
755	rs2607997	HLTF	5UR//G-4655T	--
756	rs2665796	SMARCD2	5UR//G-3364C	--
757	rs2682585	XRCC1	5UR//A-1572G	--
758	rs2727326	SMARCD2	5UR//G-2664A	--
759	rs2727327	SMARCD2	5UR//G-3070A	--
760	rs2804403	ABCC2	5UR//C-3357T	--
761	rs2805832	XPA	5UR//G-3188A	--
762	rs28372687	POLM	5UR//G-841A	--
763	rs28381715	ABCB1	5UR//T-334G	--
764	rs28382572	SMARCB1	5UR//C-3836T	--
765	rs28382580	SMARCB1	5UR//A-2457-	--
766	rs28382617	POLM	5UR//T-1584C	--
767	rs28382618	POLM	5UR//A-1410G	--
768	rs28382628	POLM	5UR//G-216A	--
769	rs28382644	POLM	E/5/C659G	G220A
770	rs28382812	CES2	E/1/C111T	I37I
771	rs28382815	CES2	E/2/C406T	R136*
772	rs28419190	SMARCAD1	5UR//T-2796C	--

773	rs2853533	TYMS	I/1/G117C	--
774	rs2853742	TYMS	5UR//T-176C	--
775	rs2854495	XRCC1	5UR//C-2834A	--
776	rs28565268	POLB	5UR//T-4791C	--
777	rs28570299	FPGS	5UR//A-1540T	--
778	rs28712867	TK1	5UR//C-3689T	--
779	rs2877173	SMARCB1	5UR//G-3584A	--
780	rs2892547	HMGB1	5UR//C-3393A	--
781	rs2917667	NQO1	5UR//A-3244G	--
782	rs2942570	DPYS	5UR//T-2427C	--
783	rs2959024	DPYS	5UR//T-493G	--
784	rs2959025	DPYS	5UR//A-902G	--
785	rs2959026	DPYS	5UR//A-3374G	--
786	rs2959027	DPYS	5UR//A-4439G	--
787	rs2964584	ATOX1	5UR//T-4667C	--
788	rs2992904	ABCC4	5UR//A-4485C	--
789	rs304731	XRCC1	5UR//T-3229C	--
790	rs3057854	SMARCA2	5UR//--745TATTTTT	--
791	rs3106134	SMARCAD1	5UR//A-1102G	--
792	rs3118106	SMARCA1	5UR//G-2060C	--
793	rs3131275	SMARCA1	5UR//A-2539G	--
794	rs3136038	ERCC4	5UR//C-643T	--
795	rs3136227	MSH6	5UR//C-613A	--
796	rs3136716	POLB	I/1/C159G	--
797	rs3172297	MLH1	5UR//T-2608C	--

798	rs3176320	CDKN1A	I/1/A213G	--
799	rs3176323	CDKN1A	I/1/T133C	--
800	rs3176628	XPA	5UR//G-845A	--
801	rs3176629	XPA	5UR//G-383A	--
802	rs3204953	REV3L	E/31/C9190T	V3064I
803	rs3212929	ERCC1	5UR//C-450A	--
804	rs3212935	ERCC1	E/1/T-143C	--
805	rs3212936	ERCC1	E/1/--78C	--
806	rs3213138	E2F1	5UR//A-322G	--
807	rs3213143	E2F1	E/1/G111A	S37S
808	rs3213174	E2F1	E/6/G932T	T311N
809	rs3213177	E2F1	E7/3UTR /C5A	--
810	rs3213180	E2F1	E7/3UTR /G914C	--
811	rs3213236	XRCC1	5UR//--1739G	--
812	rs3213246	XRCC1	E/1/G-64A	--
813	rs3218602	REV3L	E/31/A9108G	Y3036Y
814	rs3218655	POLM	E/1/C102A	L34L
815	rs3218657	POLM	E/2/C351T	V117V
816	rs322102	TDG	I/1/C94G	--
817	rs322104	TDG	5UR//G-472A	--
818	rs330792	MSH6	5UR//A-1646C	--
819	rs33934538	SMARCA2	5UR//A-2071-	--
820	rs34009709	TDG	5UR//G-2327A	--
821	rs34067256	TP53	5UR//C-1455G	--
822	rs34144509	WDR7	5UR//T-1451C	--

823	rs34213602	FDXR	I/1/C147G	--
824	rs34248325	SMARCB1	5UR//G-711A	--
825	rs34428341	CES1	5UR//C-1005T	--
826	rs34484367	SMARCE1	5UR//C-2083G	--
827	rs34547608	UGT1A1	5UR//T-90C	--
828	rs34547779	FDXR	5UR//G-667C	--
829	rs34800257	MT2A	5UR//T-1478C	--
830	rs34976170	SMARCB1	5UR//C-482G	--
831	rs34994762	MTHFR	5UR//A-4163G	--
832	rs35033646	FDXR	E12/3UTR /G139T	--
833	rs35072974	FDXR	E/8/G743A	P248L
834	rs35263175	SLC22A7	E/1/T222C	D74D
835	rs35395489	MT2A	5UR//A-561G	--
836	rs35448124	ERCC4	5UR//TGTG-4441-	--
837	rs35466868	ERBB2	5UR//C-13786T	--
838	rs35634719	FDXR	5UR//TG-1250-	--
839	rs35665780	UGT1A1	5UR//C-620T	--
840	rs35880761	FDXR	5UR//A-1824G	--
841	rs35891829	TP53	5UR//A-2059C	--
842	rs35960304	RRM1	5UR//C-3341T	--
843	rs35988004	MT1A	5UR//T-1668-/A	--
844	rs36010696	FDXR	5UR//A-1108T	--
845	rs36106739	FDXR	I/1/T292C	--
846	rs36124867	TYMS	5UR//A-4424C	--
847	rs36204705	FPGS	5UR//G-1011T	--

848	rs36209093	GSTM1	5UR//C-654T	--
849	rs36211400	GSTT1	5UR//C-688G	--
850	rs36230817	SLC19A1	5UR//A-1948G	--
851	rs36233090	SOD1	5UR//C-1659G	--
852	rs3734701	SLC29A1	5UR//T-1987C	--
853	rs3735295	PMS2	I/1/G72A	--
854	rs3735296	PMS2	5UR//G-66C	--
855	rs3737965	MTHFR	5UR//G-335A	--
856	rs3747802	ABCB1	5UR//A-21G	--
857	rs3750187	DPYS	5UR//G-3197A	--
858	rs3750747	ERCC6	I/1/G156C	--
859	rs3750994	RRM1	5UR//T-2453G	--
860	rs3750996	RRM1	5UR//A-2723G	--
861	rs3755141	SMARCAL1	5UR//C-1325T	--
862	rs3755142	SMARCAL1	5UR//T-1647C	--
863	rs376184	SMARCD1	5UR//T-4942C	--
864	rs3763505	UPP1	E/1/G-363A	--
865	rs3787037	FXYD3	5UR//A-1925G	--
866	rs3787039	FXYD3	5UR//A-2261G	--
867	rs3794050	RRM1	5UR//A-4023G	--
868	rs3806573	SMARCAL1	5UR//A-551G	--
869	rs3809159	SMUG1	5UR//G-1270C	--
870	rs3814270	ABCC4	5UR//G-639A	--
871	rs3826317	FDXR	5UR//C-491T	--
872	rs3826992	FXYD3	5UR//G-2347C	--

873	rs3829965	CDKN1A	5UR//A-1976G	--
874	rs3829967	CDKN1A	5UR//T-1528C	--
875	rs3831222	SLC29A1	5UR//G-2445-	--
876	rs406113	GPX6	E/1/A39C	F13L
877	rs4124874	UGT1A1	5UR//T-3259G	--
878	rs412543	GSTM1	5UR//G-497C	--
879	rs41275676	SMARCAD1	E/2/A-6G	--
880	rs41294988	MSH6	E/1/A38C	K13T
881	rs4135036	TDG	5UR//T-562C	--
882	rs4145763	UCK2	5UR//C-1645T	--
883	rs4147563	GSTM1	5UR//C-338T	--
884	rs4147581	GSTP1	I/1/C-20G	--
885	rs4148727	ABCB1	5UR//A-201G	--
886	rs4151702	CDKN1A	5UR//G-498C	--
887	rs423143	FGFR4	5UR//G-4265A	--
888	rs4253003	ERCC6	5UR//C-137T	--
889	rs4253006	ERCC6	E/1/C-21T	--
890	rs4253046	ERCC6	E/5/T1274G	D425A
891	rs4399719	UGT1A1	5UR//T-2457G	--
892	rs4401102	MPO	5UR//C-2540T	--
893	rs442767	DHFR	5UR//G-695T	--
894	rs453544	DHFR	5UR//G-1008A	--
895	rs45498791	MTHFR	5UR//C-479A	--
896	rs45546035	MTHFR	E/3/G276A	D92D
897	rs45557639	FGFR4	5UR//T-3458C	--

898	rs45585938	FGFR4	5UR//A-3570G	--
899	rs4647201	MLH1	5UR//C-1487A	--
900	rs4647203	MLH1	5UR//G-794A	--
901	rs4681481	HLTF	5UR//T-2502C	--
902	rs4711460	CDKN1A	5UR//C-4358A	--
903	rs4714003	CDKN1A	5UR//C-4226T	--
904	rs4724280	POLM	5UR//G-2496A	--
905	rs4741636	SMARCA2	5UR//T-1761C	--
906	rs4759344	SMUG1	5UR//A-357G	--
907	rs477415	WDR7	I/1/A292T	--
908	rs4784701	MT1A	5UR//T-1910G	--
909	rs4806085	FXYD3	5UR//T-4784C	--
910	rs4806087	FXYD3	5UR//G-3524A	--
911	rs4806088	FXYD3	5UR//G-3458A	--
912	rs4846054	MTHFR	5UR//C-3114T	--
913	rs485365	WDR7	5UR//G-3183A	--
914	rs487140	WDR7	5UR//G-3339A	--
915	rs492095	FDXR	E/1/A-9G	--
916	rs4987706	BCL2	I/1/T42C	--
917	rs501415	WDR7	I/1/A35G	--
918	rs507964	SLC29A1	5UR//T-4905G	--
919	rs535437	WDR7	5UR//G-1238A	--
920	rs559998	WDR7	5UR//G-1592T	--
921	rs561762	WDR7	5UR//C-1764T	--
922	rs572362	FDXR	5UR//T-870G	--

923	rs6003880	SMARCB1	5UR//C-219T	--
924	rs6120343	E2F1	5UR//G-3228A	--
925	rs613653	WDR7	5UR//T-4204C	--
926	rs6141997	E2F1	5UR//T-2621C	--
927	rs6151599	DHFR	5UR//A-231G	--
928	rs6151600	DHFR	5UR//T-907C	--
929	rs621018	WDR7	5UR//A-443G	--
930	rs628047	WDR7	5UR//C-3276G	--
931	rs632427	WDR7	5UR//C-703T	--
932	rs6440583	HLTF	5UR//C-857T	--
933	rs6498485	ERCC4	5UR//G-3028A	--
934	rs6499786	CES1	5UR//A-2159G	--
935	rs6499788	CES1	5UR//T-4761A	--
936	rs6511716	SMARCA4	5UR//A-3770G	--
937	rs6580978	SMUG1	5UR//A-779G	--
938	rs662855	WDR7	5UR//G-2907T	--
939	rs6672420	RUNX3	5UR//A-34239T	--
940	rs6689902	GSTM1	5UR//A-1643C	--
941	rs6714634	UGT1A1	5UR//T-4153C	--
942	rs6798870	ABCC5	5UR//G-4622A	--
943	rs6830518	PPAT	5UR//C-4949T	--
944	rs6883528	SLCO6A1	5UR//T-1419C	--
945	rs688890	WDR7	5UR//G-288A	--
946	rs689384	WDR7	5UR//T-161C	--
947	rs689456	NQO1	5UR//C-1029T	--

948	rs6898458	SLCO6A1	5UR//G-1944A	--
949	rs690115	FDXR	5UR//C-4912T	--
950	rs6976251	PMS2	5UR//G-543C	--
951	rs7040790	SMARCA2	5UR//T-1379G	--
952	rs717378	UPP2	5UR//G-3222C	--
953	rs7196890	MT1A	5UR//C-1557A	--
954	rs7202530	CES2	5UR//T-4928A	--
955	rs7219483	SMARCE1	5UR//A-3853G	--
956	rs7277748	SOD1	E/1/A-109G	--
957	rs7289159	UPB1	5UR//G-901T	--
958	rs7292735	TYMP	5UR//A-2347G	--
959	rs7328090	ATP7B	5UR//G-2175T	--
960	rs7459020	UPP1	5UR//G-149A	--
961	rs7498748	CES1	5UR//T-163C	--
962	rs7534738	UCK2	5UR//A-1501C	--
963	rs7556417	UCK2	5UR//C-2312T	--
964	rs7582263	RRM2	5UR//C-1246A	--
965	rs7619819	ABCC5	5UR//A-3831G	--
966	rs762623	CDKN1A	5UR//G-1020A	--
967	rs762624	CDKN1A	5UR//A-898C	--
968	rs7628248	HLTF	5UR//C-3217A	--
969	rs7688482	PPAT	5UR//T-2585C	--
970	rs7753792	SLC29A1	5UR//C-1718A	--
971	rs7793905	UPP1	5UR//C-1160T	--
972	rs7927657	GSTP1	5UR//T-4297C	--

973	rs7934581	RRM1	5UR//C-3890T	--
974	rs7941648	GSTP1	5UR//C-3690T	--
975	rs7945035	GSTP1	5UR//A-3123G	--
976	rs796736	TDG	5UR//T-2448A	--
977	rs7991067	HMGB1	5UR//C-4553A	--
978	rs8068725	FDXR	5UR//A-3081G	--
979	rs8071253	TK1	5UR//G-1181A	--
980	rs8073029	FDXR	5UR//T-3265C	--
981	rs812498	TDG	5UR//T-4817C	--
982	rs8132524	SOD1	5UR//C-2443T	--
983	rs8137555	SMARCB1	5UR//T-2842C	--
984	rs8177412	GPX3	E/1/T-129C	--
985	rs8177413	GPX3	E/1/G39C	L13L
986	rs8191439	GSTP1	E/1/G-18A	--
987	rs8192443	SMARCD1	5UR//C-3479A	--
988	rs868853	ABCC4	5UR//C-1388T	--
989	rs879000	WDR7	5UR//A-4398G	--
990	rs897761	FXYD3	5UR//T-326A	--
991	rs903501	ERBB2	5UR//T-16760C	--
992	rs9311149	MLH1	5UR//C-4803A	--
993	rs9333500	POLH	E/1/G-200T	--
994	rs9333500	XPO5	5UR//G-316T	--
995	rs9357436	SLC29A1	5UR//G-4521A	--
996	rs961077	UMPS	5UR//C-4613T	--
997	rs9630729	SMARCE1	5UR//T-3757C	--

998	rs9673491	MT1A	5UR//G-3655T	--
999	rs9674227	MT1A	5UR//A-3590C	--
1000	rs9890046	ABCC3	I/1/C369G	--
1001	rs9927585	CES1	5UR//G-4382A	--
1002	rs9947507	TYMS	5UR//C-555T	--
1003	rs17222547	ABCC2	E/22/C2901A	Y967*
1004	rs2066523	SMARCAL1	E/11/G1945A	R649*
1005	rs2291078	UMPS	E/4/T1050A	C350*
1006	rs3201997	ABCG2	E/9/C1000A	E334*
1007	rs5023780	CES1	E/3/G310A	R104*
1008	rs596909	TYMS	E/4/G470T	L157*
1009	rs1003355	ABCC3	E/12/C1583G	A528G
1010	rs10055840	SLCO6A1	E/12/G1961C	T654R
1011	rs10073333	SLCO6A1	E/9/G1579C	P527A
1012	rs10091081	POLB	E/8/G431C	G144A
1013	rs1042709	UGT1A1	E/5/G1531C	A511P
1014	rs1050101	SMARCD3	E/5/G508A	P170S
1015	rs1061017	ABCG2	E/5/G496C	Q166E
1016	rs1061018	ABCG2	E/6/A623G	F208S
1017	rs10964468	SMARCA2	E/2/G-5A	--
1018	rs10983315	XPA	E/3/C289T	V97I
1019	rs1132543	TK1	E/5/C442T	V148M
1020	rs1138272	GSTP1	E/6/C341T	A114V
1021	rs11553892	GSTP1	E/7/C526A	L176M
1022	rs11555797	SMARCAL1	E/2/G341A	T114M

1023	rs11568587	ABCC3	E/22/-3051-	--
1024	rs11568591	ABCC3	E/27/G3890A	R1297H
1025	rs11568599	ABCC3	E/15/-1926-	--
1026	rs11568658	ABCC4	E/5/C559A	G187W
1027	rs11568669	ABCC4	E/11/T1492C	K498E
1028	rs11568705	ABCC4	E/9/G1208A	P403L
1029	rs11656685	ABCC3	E/31/C4538A	A1513D
1030	rs11708427	ABCC5	3DR//C1158G	--
1031	rs11722476	SMARCAD1	E/7/G740A	S247N
1032	rs11723410	SMARCAD1	E/3/C197T	S66F
1033	rs11954456	FGFR4	E/6/C825G	S275R
1034	rs11971829	UPP1	E/3/C11T	T4M
1035	rs1202183	ABCB1	E/5/T131C	N44S
1036	rs12103928	SMARCE1	E/5/T183G	K61N
1037	rs12367528	TDG	E/10/C1136A	P379H
1038	rs12388502	SMARCA1	E/17/C2113G	E705Q
1039	rs12678588	POLB	E/7/G410A	R137Q
1040	rs12682945	UCK1	E/7/A785G	L262P
1041	rs12686275	FPGS	E/14/T1160A	V387D
1042	rs12808005	RRM1	E/7/A536C	H179P
1043	rs12928616	ERCC4	E/11/C2240T	S747F
1044	rs12928650	ERCC4	E/11/C2303T	S768F
1045	rs13079661	SMARCC1	E/14/T1357A	S453C
1046	rs13091100	HLTF	E/3/A248C	V83G
1047	rs13308178	POLM	E/10/C1357G	G453R

1048	rs1332018	GSTM3	E/1/G-63T	--
1049	rs13400205	RRM2	E/4/T320G	G107V
1050	rs1695	GSTP1	E/5/A313G	I105V
1051	rs17150488	SLCO6A1	E/7/T1142C	K381R
1052	rs17216317	ABCC2	E/28/C3872T	P1291L
1053	rs17222674	ABCC2	E/8/A998G	D333G
1054	rs17510963	REV3L	E/14/A6225C	I2075M
1055	rs17539588	REV3L	E/13/C2885T	R962Q
1056	rs17539616	REV3L	E/13/G4015T	P1339T
1057	rs17539692	REV3L	E/14/T6044A	E2015V
1058	rs17843776	UMPS	E/1/A88G	R30W
1059	rs17843819	UMPS	E/3/T859C	E287K
1060	rs17880492	GPX2	E/2/G436A	R146C
1061	rs17882252	TP53	E/10/C1015T	E339K
1062	rs1799792	ERCC2	E/8/G601A	H201Y
1063	rs1799802	ERCC4	E/7/C1135T	P379S
1064	rs1800067	ERCC4	E/8/G1244A	R415Q
1065	rs1800068	ERCC4	E/8/G1727C	R576T
1066	rs1800124	ERCC4	E/11/A2624G	E875G
1067	rs1800152	MSH2	E/12/T1917G	H639Q
1068	rs1800938	MSH6	E/4/A660C	E220D
1069	rs1801201	ERBB2	E/17/A1960G	I654V
1070	rs1801244	ATP7B	E/3/C1366G	V456L
1071	rs1801266	DPYD	E/7/G703A	R235W
1072	rs1803687	GSTM3	E/6/C384G	K128N

1073	rs1805318	PMS2	E/11/T1789A	T597S
1074	rs1805322	PMS2	E/8/G830T	T277K
1075	rs1805323	PMS2	E/11/G1454T	T485K
1076	rs1805324	PMS2	E/11/C1866T	M622I
1077	rs1966265	FGFR4	E/1/G28A	V10I
1078	rs2020908	MSH6	E/4/C1186G	L396V
1079	rs2020955	ERCC4	E/10/T1984C	S662P
1080	rs2020956	ERCC4	E/11/G2735A	G912E
1081	rs2020959	ERCC4	E/11/C2169A	C723*
1082	rs2020961	ERCC4	E/3/C503T	A168V
1083	rs2066472	MTHFR	E/2/C203T	R68Q
1084	rs2066522	SMARCAL1	E/4/C945G	Q315H
1085	rs2066524	SMARCAL1	E/2/G127A	C43R
1086	rs2227291	ATP7A	E/10/G2299C	V767L
1087	rs2227963	GSTM5	E/7/G536A	L179P
1088	rs2228006	PMS2	E/11/T1621C	E541K
1089	rs2228529	ERCC6	E/21/T4238C	Q1413R
1090	rs2229107	ABCB1	E/27/A3421T	S1141T
1091	rs2229361	HLTF	E/21/C2456T	R819H
1092	rs2229996	APC	E/16/C4487G	T1496S
1093	rs2231137	ABCG2	E/2/C34T	V12M
1094	rs2233915	SLC31A1	E/2/C73G	P25A
1095	rs2233916	SLC31A1	E/4/C365G	T122S
1096	rs2234935	ATP7A	E/9/T2006C	I669T
1097	rs2234953	GSTT1	E/4/C517T	E173K

1098	rs2235036	ABCB1	E/16/C1795T	A599T
1099	rs2235039	ABCB1	E/21/C2401T	V801M
1100	rs2266633	GSTT1	E/4/C421T	D141N
1101	rs2271336	SMARCAL1	E/13/C2225T	Y742C
1102	rs2274974	MTHFR	E/11/C1697T	G566E
1103	rs2277447	ATP7B	E/7/C2029T	E677K
1104	rs2296212	SMARCA2	E/32/C4584G	D1528E
1105	rs2305868	HLTF	E/8/T932C	N311S
1106	rs2307167	XRCC1	E/15/C1676T	R559Q
1107	rs2307184	XRCC1	E/13/G1454T	S485Y
1108	rs2307191	XRCC1	E/5/G482A	P161L
1109	rs2307227	CES1	E/5/G609T	D203E
1110	rs2307240	CES1	E/2/C224T	S75N
1111	rs2307456	POLH	E/5/G626T	G209V
1112	rs2388544	HMGB1	E/3/G293A	P98L
1113	rs25474	XRCC1	E/14/G1541A	P514L
1114	rs25491	XRCC1	E/9/G925A	P309S
1115	rs2632398	SMARCAD1	E/4/C418T	R140C
1116	rs2682557	XRCC1	E/16/T1726A	Y576N
1117	rs28364274	ABCB1	E/29/C3751T	V1251I
1118	rs28381801	ABCB1	E/2/T-42C	--
1119	rs28381902	ABCB1	E/15/C1696T	E566K
1120	rs28381967	ABCB1	E/22/T2506C	I836V
1121	rs28382653	POLM	E/6/C736A	V246F
1122	rs28382661	POLM	E/11/G1450A	L484F

1123	rs28401798	ABCB1	E/26/G3151C	P1051A
1124	rs28563878	CES1	E/1/A34C	S12A
1125	rs3103135	SMARCAD1	E/14/A1765C	N589H
1126	rs3113842	SMARCAD1	E/12/T1612C	Y538H
1127	rs3116448	ABCG2	E/7/A742G	S248P
1128	rs3136334	MSH6	E/4/C1867G	P623A
1129	rs3136389	SMUG1	E/4/G313A	R105W
1130	rs3188420	ERCC1	E/2/G230T	P77H
1131	rs3212057	XRCC3	E/5/C281T	R94H
1132	rs3212977	ERCC1	E/8/C796T	A266T
1133	rs3213172	E2F1	E/5/C755T	R252H
1134	rs3213173	E2F1	E/5/C826T	V276M
1135	rs3213176	E2F1	E/7/C1177T	G393S
1136	rs3218572	REV3L	E/13/T4406G	Q1469P
1137	rs3218578	REV3L	E/13/T3850G	T1284P
1138	rs3218579	REV3L	E/10/T1190G	Q397P
1139	rs3218582	REV3L	E/13/G4727A	S1576L
1140	rs3218585	REV3L	E/13/C5137T	D1713N
1141	rs3218592	REV3L	E/26/C8285T	R2762Q
1142	rs3218593	REV3L	E/13/A2078G	M693T
1143	rs3218595	REV3L	E/13/C3927A	Q1309H
1144	rs3218600	REV3L	E/13/G3659A	S1220L
1145	rs3218604	REV3L	E/14/C5767G	G1923R
1146	rs3218606	REV3L	E/14/C5909T	R1970H
1147	rs33974176	APC	E/16/C2608T	P870S

1148	rs34110964	UPB1	E/9/C1019A	A340D
1149	rs34138361	FGFR4	E/11/C1652T	S551F
1150	rs34157245	APC	E/16/G5645C	R1882T
1151	rs34284947	FGFR4	E/10/G1466A	R489Q
1152	rs34354111	FPGS	E/15/G1433C	S478T
1153	rs34447156	NQO1	E/5/C693G	Q231H
1154	rs34622270	HLTF	E/20/T2284C	I762V
1155	rs34628871	MPO	E/1/A-65G	--
1156	rs34825130	GPX6	E/2/A157G	Y53H
1157	rs35003977	UGT1A1	E/1/T674G	V225G
1158	rs35102176	FDXR	E/7/G624C	C208W
1159	rs35163653	TP53	E/6/C649T	V217M
1160	rs35338630	MLH1	E/9/C790G	H264D
1161	rs35394555	GPX6	E/4/C408G	E136D
1162	rs35578165	FXYD3	E/6/G118A	G40S
1163	rs35658392	GPX6	E/5/G469A	P157S
1164	rs35660143	FDXR	E/10/G1034A	T345M
1165	rs35667202	UPP2	E/8/G674T	R225L
1166	rs35670089	MPO	E/11/G1810A	R604C
1167	rs35671174	CDC2	E/6/A665G	K222R
1168	rs35675573	POLH	E/8/C986T	T329I
1169	rs35702888	MPO	E/12/C2047G	E683Q
1170	rs35717727	MSH6	E/8/G3700C	E1234Q
1171	rs35737219	MTHFR	E/12/G1958A	T653M
1172	rs35993958	TP53	E/10/C1079G	G360A

1173	rs36027551	DPYS	E/3/G541A	R181W
1174	rs36040909	SLC22A7	E/6/C973T	R325W
1175	rs3740071	ABCC2	E/20/G2677C	E893Q
1176	rs3740072	ABCC2	E/17/A2153G	N718S
1177	rs3765534	ABCC4	E/18/C2269T	E757K
1178	rs376618	FGFR4	E/3/C407T	P136L
1179	rs3772406	SMARCC1	E/28/G3224T	P1075H
1180	rs3772809	UMPS	E/6/A1336G	H446Y
1181	rs377860	APC	E/16/A4108C	K1370Q
1182	rs3793510	SMARCA2	E/29/G4247C	G1416A
1183	rs3826192	CES1	E/2/C112T	V38I
1184	rs41295268	MSH6	E/4/G1403A	R468H
1185	rs41295270	MSH6	E/4/C1739T	S580L
1186	rs41306702	FPGS	E/2/C103T	R35W
1187	rs41318029	ABCC2	E/21/G2761A	G921S
1188	rs4135038	TDG	E/1/G-169A	--
1189	rs4135113	TDG	E/5/G595A	G199S
1190	rs4148323	UGT1A1	E/1/G211A	G71R
1191	rs4148460	ABCC4	E/4/A511C	C171G
1192	rs4150522	ERCC3	E/14/A2203G	S735P
1193	rs41540513	ERCC1	5UR//G-320A	--
1194	rs41542214	MLH1	E/18/C2065A	Q689K
1195	rs41549213	ERCC6	E/7/C1670T	R557H
1196	rs41552412	ERCC4	E/8/C1563G	S521R
1197	rs41557814	ERCC4	E/8/C1429T	R477W

1198	rs41557921	ERCC6	E/15/C2803G	D935H
1199	rs41559922	ERCC2	E/14/A1343G	F448S
1200	rs4253047	ERCC6	E/5/C1337T	G446D
1201	rs4253206	ERCC6	E/17/T3005C	Y1002C
1202	rs4253219	ERCC6	E/19/C3965A	G1322V
1203	rs4253227	ERCC6	E/21/C4114T	G1372R
1204	rs4253230	ERCC6	E/21/G4322A	T1441I
1205	rs4338942	SMARCAL1	E/2/T569G	V190G
1206	rs45441199	ABCC2	E/23/T3107C	I1036T
1207	rs45458701	SLC29A1	E/12/G1171A	E391K
1208	rs45462493	ABCC2	E/7/A736C	M246L
1209	rs45477596	ABCC4	3DR//C12523T	--
1210	rs45496998	MTHFR	E/10/G1555A	R519C
1211	rs45504892	ABCC4	3DR//C20231A	--
1212	rs45573936	SLC29A1	E/7/T647C	I216T
1213	rs45589337	DPYD	E/8/T775C	K259E
1214	rs45617731	ABCC3	E/8/G941T	S314I
1215	rs458017	REV3L	E/13/T3467C	Y1156C
1216	rs4705693	APC	E/16/G7567T	A2523S
1217	rs4826245	ATP7A	E/21/G4048A	E1350K
1218	rs4851	GPX4	E/7/C680T	S227L
1219	rs4986866	CDKN1A	E/2/C11T	P4L
1220	rs4986867	CDKN1A	E/2/C189A	F63L
1221	rs4986949	GSTP1	E/6/G439T	D147Y
1222	rs4997557	CYP2A6	E/6/G881C	T294S

1223	rs520611	FDXR	E/2/C120A	Q40H
1224	rs5959130	ATP7A	E/22/G4201C	V1401L
1225	rs598078	WDR7	E/3/A235C	K79Q
1226	rs6446261	GPX1	E/2/C580T	A194T
1227	rs6710480	UPP2	E/3/G30T	R10S
1228	rs6941583	POLH	E/11/A1939T	M647L
1229	rs7020514	SMARCA2	E/8/T1362G	S454R
1230	rs7080681	ABCC2	E/9/G1058A	R353H
1231	rs728619	MSH6	E/4/A1613C	Y538S
1232	rs7356934	REV3L	E/25/G8036A	P2679L
1233	rs7439869	SMARCAD1	E/9/T902C	A301V
1234	rs7483	GSTM3	E/8/C670T	V224I
1235	rs7561584	UPP2	E/5/A232C	M78L
1236	rs769188	GPX5	E/3/C253G	L85V
1237	rs8177445	GPX3	E/4/T382C	F128L
1238	rs8192730	CYP2A6	E/8/C1257G	E419D
1239	rs8192924	CES2	E/5/G809A	R270H
1240	rs927344	ABCC2	E/2/A116T	F39Y
1241	rs9282571	ABCG2	E/14/A1711T	F571I
1242	rs9296419	POLH	E/11/C1433T	T478M
1243	rs9333555	POLH	E/11/A1783G	M595V
1244	rs1042858	RRM1	E/19/G2232A	A744A
1245	rs1048977	CDA	E/4/C435T	T145T
1246	rs1050102	SMARCD3	E/8/G828A	H276H
1247	rs1056806	GSTM1	E/7/C528T	D176D

1248	rs1065767	TK1	E/7/G720A	A240A
1249	rs10964471	SMARCA2	E/2/G177A	T59T
1250	rs11100790	SMARCA5	E/3/T282C	Y94Y
1251	rs11553301	UCK2	E/4/G408A	G136G
1252	rs11568695	ABCC4	3DR//C51485T	--
1253	rs11568704	ABCC4	3DR//C61070T	--
1254	rs11682453	UGT1A1	E/4/C1279T	L427L
1255	rs11786893	GGH	E/2/C174T	A58A
1256	rs11840224	ATP7B	E/13/G2967A	D989D
1257	rs1189466	ABCC4	3DR//A21484G	--
1258	rs1200937	CES2	E/1/G-140C	--
1259	rs12532895	PMS2	E/4/G288A	A96A
1260	rs13288443	SMARCA2	E/11/A1827G	P609P
1261	rs13306555	MTHFR	E/6/G906A	A302A
1262	rs13427563	ERCC3	E/5/C615T	E205E
1263	rs13428173	ERCC3	E/11/G1740A	I580I
1264	rs17090346	WDR7	E/15/C2542T	L848L
1265	rs17216296	ABCC2	E/30/C4242T	H1414H
1266	rs17510914	REV3L	E/13/T4650C	P1550P
1267	rs17849090	PTEN	E/7/T723C	L241L
1268	rs1800144	MLH1	E/4/A375G	A125A
1269	rs1800150	MSH2	E/2/G219A	K73K
1270	rs1800369	TP53	E/2/G63A	D21D
1271	rs1800932	MSH6	E/2/A276G	P92P
1272	rs1800935	MSH6	E/3/T540C	D180D

1273	rs1801018	BCL2	E/2/T21C	T7T
1274	rs1801019	UMPS	E/3/G638C	--
1275	rs1801248	ATP7B	E/13/C3045T	L1015L
1276	rs1970951	GPX7	E/2/T237C	F79F
1277	rs2020913	MSH6	E/4/T2253C	N751N
1278	rs2020953	ERCC4	E/11/A2463G	P821P
1279	rs2020958	ERCC4	E/9/A1884G	E628E
1280	rs2066527	SMARCAL1	E/11/T2070C	N690N
1281	rs2228544	ERCC3	E/8/C1119T	Q373Q
1282	rs2229993	APC	E/16/G6921A	S2307S
1283	rs2229997	APC	E/16/C5250G	V1750V
1284	rs2230761	UPP1	E/7/A606G	T202T
1285	rs2232867	UPB1	E/7/C846T	F282F
1286	rs2232870	UPB1	E/10/T1086C	Y362Y
1287	rs2288845	SMARCA4	E/9/C1557T	N519N
1288	rs2307174	XRCC1	E/3/C150T	E50E
1289	rs2307189	XRCC1	E/2/G126T	T42T
1290	rs2307460	POLH	E/6/C678T	A226A
1291	rs28381867	ABCB1	E/9/C738T	A246A
1292	rs28382610	GPX5	E/5/C633T	I211I
1293	rs28382827	CES2	E/12/C1791T	L597L
1294	rs28997580	SMARCA4	E/16/C2388T	L796L
1295	rs28997582	SMARCA4	E/29/C4053T	D1351D
1296	rs3136804	POLB	E/13/C888T	Y296Y
1297	rs3212045	XRCC3	E/4/G132A	P44P

1298	rs3218577	REV3L	E/27/C8367A	L2789L
1299	rs34219015	WDR7	E/22/C3714A	I1238I
1300	rs34237683	RRM2	E/1/C87T	R29R
1301	rs34308410	SMARCC2	E/13/T1158C	E386E
1302	rs34312619	MSH2	E/2/C336A	S112S
1303	rs34474865	HLTF	E/16/T1740C	R580R
1304	rs34566456	MLH1	5UR//G-532C	--
1305	rs35013010	DPYS	E/6/A1062G	D354D
1306	rs35043160	APC	E/16/A7704G	G2568G
1307	rs35051203	SMARCD3	E/13/C1422T	L474L
1308	rs35182583	ERCC6	E/10/C2082T	P694P
1309	rs35225190	MLH1	E/7/A552T	S184S
1310	rs35420817	PPAT	E/9/T1026C	P342P
1311	rs35464006	ERBB2	5UR//G-15393C	--
1312	rs35642130	MSH6	E/5/G3354A	E1118E
1313	rs35653697	MTHFR	E/9/C1476T	P492P
1314	rs35756610	ERCC6	E/18/T3774C	K1258K
1315	rs35908749	MLH1	E/9/G702A	E234E
1316	rs3737967	MTHFR	E12/3UTR /G3288A	--
1317	rs3916876	ERCC2	E/18/G1737A	V579V
1318	rs41280126	ABCC3	E/16/A2043G	L681L
1319	rs4135119	TDG	E/8/C795T	L265L
1320	rs4135120	TDG	E/8/C867T	Y289Y
1321	rs41557516	REV3L	E/30/G8874A	P2958P
1322	rs4252610	ERBB2	E/2/G99A	L33L

1323	rs4252655	ERBB2	E/25/C3078T	P1026P
1324	rs4252656	ERBB2	E/27/G3531A	K1177K
1325	rs4253013	ERCC6	E/2/C411T	L137L
1326	rs4253027	ERCC6	E/3/T528C	R176R
1327	rs4253044	ERCC6	E/5/A885G	A295A
1328	rs4253210	ERCC6	E/18/A3534G	F1178F
1329	rs446382	FGFR4	E/2/T162G	R54R
1330	rs452885	FGFR4	E/5/C702T	R234R
1331	rs455732	REV3L	E/13/C4290T	V1430V
1332	rs458486	REV3L	E/13/T2706C	G902G
1333	rs4647256	MLH1	E/6/C474T	N158N
1334	rs4807542	GPX4	5UR//G-570A	--
1335	rs507082	SMARCC1	E/5/G532A	L178L
1336	rs6670886	DPYD	E/6/C525T	S175S
1337	rs6823404	SMARCAD1	E/15/C1839T	D613D
1338	rs689453	NQO1	E/2/C72T	E24E
1339	rs6947955	UPP1	E/2/C-43T	--
1340	rs6972869	PMS2	E/11/A1557G	Y519Y
1341	rs7136420	SMARCC2	E/5/T438C	P146P
1342	rs7275	SMARCA4	E/34/T4887C	D1629D
1343	rs7636910	ABCC5	3DR//T2025C	--
1344	rs7899457	ABCC2	E/29/C4110T	L1370L
1345	rs8182267	ERBB2	5UR//G-4942A	--
1346	rs8187630	SLC29A1	E/3/G84A	P28P
1347	rs8187706	ABCC2	E/31/G4410A	E1470E

1348	rs8187707	ABCC2	E/31/C4488T	H1496H
1349	rs9105	SMARCA4	E/32/C4584T	D1528D
1350	rs939336	ABCC5	3DR//A16007G	--
1351	rs1537516	MTHFR	E12/3UTR /G2876A	--
1352	rs17885803	TP53	5UR//C-1564T	--
1353	rs17886250	TP53	I/1/C148T	--
1354	rs2119342	HLTF	E25/3UTR /T386C	--
1355	rs28399438	CYP2A6	I/1/A-34C	--
1356	rs2856857	MPO	I/1/G-13A	--
1357	rs3176626	XPA	5UR//A-951C	--
1358	rs34957864	HLTF	I/1/C235T	--
1359	rs3829963	CDKN1A	5UR//C-2100A	--
1360	rs3890213	CES2	5UR//C-1547T	--
1361	rs408626	DHFR	5UR//T-332C	--
1362	rs4987707	BCL2	I/1/C-87T	--
1363	rs11568589	ABCC3	E/28/G4008A	L1336L
1364	rs2307083	SMARCD1	E/4/A423G	V141V
1365	rs3547	XRCC1	E/17/T1896C	Q632Q
1366	rs1042482	DPYD	E23/3UTR /C573T	--
1367	rs1061388	REV3L	E32/3UTR /A925C	--
1368	rs11591427	PTEN	E9/3UTR /A2821T	--
1369	rs1803541	ERCC3	E15/3UTR /C259T	--
1370	rs2584622	SMARCD2	E13/3UTR /G131A	--
1371	rs35180794	GPX7	E3/3UTR /T162-	--
1372	rs41436046	FPGS	E15/3UTR /TT442-	--

1373	rs4150525	ERCC3	E15/3UTR /T177C	--
1374	rs4252661	ERBB2	E27/3UTR /T591C	--
1375	rs45616134	SMARCA2	E33/3UTR /T698C	--
1376	rs4987864	BCL2	3DR//C193453A	--
1377	rs4987865	BCL2	3DR//C193630T	--
1378	rs4987868	BCL2	3DR//G194378T	--
1379	rs5030760	RRM2	E10/3UTR /T893C	--
1380	rs5031031	GSTP1	E7/3UTR /A56G	--
1381	rs7337469	HMGB1	E5/3UTR /T2156C	--
1382	rs975922	HLTF	E25/3UTR /A1326G	--
1383	rs9955129	WDR7	E27/3UTR /T943C	--
1384	rs1042927	RRM1	E19/3UTR /C316A	--
1385	rs1047619	GPX7	E3/3UTR /A315G	--
1386	rs1047635	GPX7	E3/3UTR /C435A	--
1387	rs10981708	SLC31A1	E5/3UTR /C3287T	--
1388	rs11151988	WDR7	E27/3UTR /T1272G	--
1389	rs11610906	TDG	E10/3UTR /C1585G	--
1390	rs12106470	SLC19A1	E6/3UTR /G701T	--
1391	rs12329692	SLC19A1	E6/3UTR /G172A	--
1392	rs16948421	TYMS	E7/3UTR /G398A	--
1393	rs16950472	ABCC4	3DR//A75520G	--
1394	rs16956880	TP53	E11/3UTR /C205T	--
1395	rs17135042	APC	E16/3UTR /T1050C	--
1396	rs17225060	MSH2	E16/3UTR /A226G	--
1397	rs2230303	GPX3	E5/3UTR /T138G	--

1398	rs2735347	PTEN	E9/3UTR /T957G	--
1399	rs28382578	SMARCB1	5UR//G-3038A	--
1400	rs3176359	CDKN1A	E3/3UTR /G1165A	--
1401	rs3733326	PPAT	E11/3UTR /G284C	--
1402	rs3742106	ABCC4	3DR//A74234C	--
1403	rs3772810	UMPS	E6/3UTR /A28G	--
1404	rs397768	APC	E16/3UTR /G1753A	--
1405	rs4835	FXYD3	E9/3UTR /A173T	--
1406	rs699517	TYMS	E7/3UTR /C19T	--
1407	rs8177450	GPX3	E5/3UTR /A255G	--
1408	rs8177452	GPX3	E5/3UTR /A608G	--
1409	rs8192925	CES2	E12/3UTR/A69G	--
1410	rs873652	FGFR4	E16/3UTR /A26T	--
1411	rs9468385	GPX6	E5/3UTR /C457T	--
1412	rs9516521	ABCC4	3DR//T74903C	--
1413	rs9835477	SMARCC1	E28/3UTR /T412G	--
1414	rs10106	FPGS	E15/3UTR /T192C	--
1415	rs1016860	BCL2	3DR//C190113T	--
1416	rs1042710	UGT1A1	E5/3UTR /A3G	--
1417	rs1045411	HMGB1	E5/3UTR /C2262T	--
1418	rs1050569	GPX1	E1/3UTR /T695G	--
1419	rs10513202	SLC31A1	E5/3UTR /A2206G	--
1420	rs1051332	ATP7B	E17/3UTR /C1172T	--
1421	rs10513347	HLTF	E25/3UTR /T1491C	--
1422	rs10517	NQO1	E5/3UTR /A1119G	--

1423	rs1059316	GPX3	E5/3UTR /C713T	--
1424	rs10636	MT2A	E3/3UTR /G77C	--
1425	rs1065769	TK1	E7/3UTR /C105T	--
1426	rs10929303	UGT1A1	E5/3UTR /T211C	--
1427	rs11375183	SMARCC1	E28/3UTR /-898G	--
1428	rs1143245	SLC31A1	E5/3UTR /G3502C	--
1429	rs12010382	ATP7A	E23/3UTR /T1819C	--
1430	rs12189	APC	E16/3UTR /C434T	--
1431	rs12852	FXYD3	E9/3UTR /A818G	--
1432	rs13048427	SLC19A1	E6/3UTR /G23A	--
1433	rs1537514	MTHFR	E12/3UTR /G2669C	--
1434	rs16842633	UPP2	E9/3UTR /C962T	--
1435	rs16861315	HLTF	E25/3UTR /C1641T	--
1436	rs16948409	TYMS	E7/3UTR /G214T	--
1437	rs17225053	MSH2	E16/3UTR /T141G	--
1438	rs17387924	SMARCA2	E33/3UTR /G431A	--
1439	rs17511602	REV3L	E32/3UTR /T293C	--
1440	rs1787474	WDR7	E27/3UTR /T275C	--
1441	rs17880487	SOD1	E5/3UTR /C339T	--
1442	rs17881366	TP53	E11/3UTR /C328T	--
1443	rs1804447	SOD1	E5/3UTR /C2T	--
1444	rs2020906	MSH6	E10/3UTR /T85A	--
1445	rs2074954	UPP2	E9/3UTR /T123G	--
1446	rs2077360	MTHFR	E12/3UTR /A1858G	--
1447	rs2290736	SMARCD3	E13/3UTR /C65G	--

1448	rs2635723	DPYD	E23/3UTR /G466T	--
1449	rs2736630	PTEN	E9/3UTR /T1133C	--
1450	rs2790	TYMS	E7/3UTR /A89G	--
1451	rs28364275	ABCB1	E29/3UTR /A21G	--
1452	rs28364277	ABCB1	E29/3UTR /C146T	--
1453	rs28364279	ABCB1	E29/3UTR /T252G	--
1454	rs28364280	ABCB1	E29/3UTR /C316T	--
1455	rs28364281	ABCB1	E29/3UTR /A562G	--
1456	rs28364610	SMARCA5	5UR//G-130A	--
1457	rs28382662	POLM	E11/3UTR /T139C	--
1458	rs28382664	POLM	E11/3UTR /C676T	--
1459	rs3136391	SMUG1	E4/3UTR /A260G	--
1460	rs3136392	SMUG1	E4/3UTR /C334T	--
1461	rs3176358	CDKN1A	E3/3UTR /G385A	--
1462	rs3176753	XPA	E6/3UTR /A278G	--
1463	rs3176754	XPA	E6/3UTR /T464C	--
1464	rs3177111	GPX4	E7/3UTR /C140A	--
1465	rs3212116	XRCC3	E10/3UTR /A145G	--
1466	rs3212117	XRCC3	E10/3UTR /G448T	--
1467	rs3212125	XRCC3	E10/3UTR /A868C	--
1468	rs3212126	XRCC3	E10/3UTR /C1094T	--
1469	rs34874603	GPX6	E5/3UTR /A908G	--
1470	rs35091626	GPX7	E3/3UTR /A549T	--
1471	rs35481105	PTEN	E9/3UTR /A1697G	--
1472	rs35919705	TP53	E11/3UTR /G105A	--

1473	rs3744935	BCL2	3DR//C190507T	--
1474	rs3745030	WDR7	E27/3UTR /C2176G	--
1475	rs3745032	WDR7	E27/3UTR /C112T	--
1476	rs3745033	WDR7	E27/3UTR /C14T	--
1477	rs3749440	ABCC5	E7/3UTR /A633G	--
1478	rs3749444	ABCC5	3DR//C62512T	--
1479	rs3805114	ABCC5	3DR//T63819G	--
1480	rs3895070	PTEN	E9/3UTR /T320G	--
1481	rs4097504	HMGB1	E5/3UTR /T1167C	--
1482	rs41275468	MTHFR	E12/3UTR /G2594A	--
1483	rs41292780	ATP7B	E17/3UTR /C1385T	--
1484	rs41297348	ABCB1	E29/3UTR /A393G	--
1485	rs41483150	FPGS	E15/3UTR /A629C	--
1486	rs4148551	ABCC4	3DR//T74507C	--
1487	rs4148555	ABCC4	3DR//A75760T	--
1488	rs4148595	ABCC5	3DR//G63298A	--
1489	rs4150523	ERCC3	E15/3UTR /G29A	--
1490	rs4252658	ERBB2	E27/3UTR /C66T	--
1491	rs4253231	ERCC6	E21/3UTR /A53G	--
1492	rs448475	APC	E16/3UTR /C1556G	--
1493	rs45574135	MTHFR	E12/3UTR /C1070T	--
1494	rs45593641	NQO1	E5/3UTR /C380T	--
1495	rs45625835	MTHFR	E12/3UTR /C543T	--
1496	rs4968187	TP53	E11/3UTR /C485T	--
1497	rs4987843	BCL2	3DR//C189646T	--

1498	rs4987844	BCL2	3DR//T189914G	--
1499	rs4987845	BCL2	3DR//C189999T	--
1500	rs4987848	BCL2	3DR//C190271T	--
1501	rs4987850	BCL2	3DR//C190671T	--
1502	rs4987851	BCL2	3DR//C190928T	--
1503	rs4987852	BCL2	3DR//T191266C	--
1504	rs4987854	BCL2	3DR//C191561T	--
1505	rs4987856	BCL2	3DR//C191693T	--
1506	rs4987858	BCL2	3DR//A192393G	--
1507	rs4987860	BCL2	3DR//C192600T	--
1508	rs4987866	BCL2	3DR//T193866C	--
1509	rs4987867	BCL2	3DR//C194115T	--
1510	rs4987869	BCL2	3DR//T194457G	--
1511	rs6594650	APC	E16/3UTR /A1203C	--
1512	rs6650282	ABCC4	E21/3UTR /T168C	--
1513	rs6899628	POLH	E11/3UTR /C290T	--
1514	rs6919	SMARCD2	E13/3UTR /T813A	--
1515	rs701848	PTEN	E9/3UTR /T1516C	--
1516	rs7032466	SLC31A1	E5/3UTR /T2808G	--
1517	rs7048532	SMARCA2	E33/3UTR /T620C	--
1518	rs757412	SMARCE1	E11/3UTR /G110A	--
1519	rs7778745	POLM	E11/3UTR /A361G	--
1520	rs8026	SMARCAD1	E24/3UTR /A1500G	--
1521	rs8056468	NQO1	E5/3UTR /G776A	--
1522	rs8065799	TP53	E11/3UTR /T9G	--

1523	rs8330	UGT1A1	E5/3UTR /G440C	--
1524	rs8336	SMARCAD1	E24/3UTR /T925C	--
1525	rs868014	MTHFR	E12/3UTR /A1290G	--
1526	rs9516520	ABCC4	3DR//T75167C	--
1527	rs9535794	ATP7B	E17/3UTR /G1744A	--
1528	rs9590161	ABCC4	3DR//A75889G	--
1529	rs9680103	SLC19A1	E6/3UTR /C421A	--
1530	rs971	SMUG1	E4/3UTR /T422C	--
1531	rs10609062	UCK1	E7/3UTR/GTGA 426-	--
1532	rs2266636	GSTT1	E/4/C354T	V118V
1533	rs28364609	SMARCA5	5UR//T-740C	--
1534	rs28606552	SMARCA5	5UR//T-3655A	--
1535	rs3218658	POLM	E/8/C1032T	A344A
1536	rs6836313	SMARCA5	5UR//A-811C	--
