# Supplementary material for: Potentially Functional SNPs (pfSNPs) as Novel Genomic Predictors of 5-FU Response in Metastatic Colorectal Cancer Patients
Source: PLoS One. 2014 Nov 5;9(11):e111694. doi: 10.1371/journal.pone.0111694 (PMC4221105; doi:10.1371/journal.pone.0111694)
Supplement: Table S2 — The 14 markers not suitable to be genotyped by GoldenGate array and genotyped by other methods. (PDF) [file pone.0111694.s007.pdf]

Table S2. The 14 markers not suitable to be genotyped by GoldenGate array and genotyped by other methods.

#	rs No	Gene	mRNA Location	AA Change
1	rs2853542	TYMS	E/1/G-58C	--
2	rs34743033	TYMS	28 bp VNTR in 5UTR	--
3	rs16430	TYMS	6 bp InDel in 3UTR	--
4	rs3786362	TYMS	E/3/A381G	I127I
5	rs1801159	DPYD	E/13/T1627C	I543V
6	rs2297595	DPYD	E/6/T496C	M166V
7	rs11479	TYMP	E/10/G1412A	S471L
8	rs9628204	TYMP	E/7/C787T	G263R
9	rs17851631	TYMP	E/5/G585T	D195E
10	rs28931613	TYMP	E/2/C131T	R44Q
11	rs3210145	TYMP	I/6/T-2A	--
12	rs470119	TYMP	I/4/T27C	--
13	rs1061205	TYMP	E/10/G1401A	F467F
14	rs131804	TYMP	E/8/G972A	A324A
