# Supplementary material for: Potentially Functional SNPs (pfSNPs) as Novel Genomic Predictors of 5-FU Response in Metastatic Colorectal Cancer Patients
Source: PLoS One. 2014 Nov 5;9(11):e111694. doi: 10.1371/journal.pone.0111694 (PMC4221105; doi:10.1371/journal.pone.0111694)
Supplement: Table S3 — The MAF for SNPs selected for genotyping in each region based on HapMap R27 CHB. (PDF) [file pone.0111694.s008.pdf]

Table S3. The MAF for SNPs selected for genotyping in each region based on HapMap R27 CHB.

	Coding, 3' UTR and Promoter	Intron
MAF >5	331	323
MAF <5	129	74
MAF =0	201	2
Unknown	467	23
